# Lives versus Livelihoods? Perceived economic risk has a stronger association with support for COVID-19 preventive measures than perceived health risk

**DOI:** 10.1038/s41598-021-88314-4

**Published:** 2021-05-06

**Authors:** Claudia F. Nisa, Jocelyn J. Bélanger, Daiane G. Faller, Nicholas R. Buttrick, Jochen O. Mierau, Maura M. K. Austin, Birga M. Schumpe, Edyta M. Sasin, Maximilian Agostini, Ben Gützkow, Jannis Kreienkamp, Georgios Abakoumkin, Jamilah Hanum Abdul Khaiyom, Vjollca Ahmedi, Handan Akkas, Carlos A. Almenara, Mohsin Atta, Sabahat Cigdem Bagci, Sima Basel, Edona Berisha Kida, Allan B. I. Bernardo, Phatthanakit Chobthamkit, Hoon-Seok Choi, Mioara Cristea, Sára Csaba, Kaja Damnjanović, Ivan Danyliuk, Arobindu Dash, Daniela Di Santo, Karen M. Douglas, Violeta Enea, Gavan Fitzsimons, Alexandra Gheorghiu, Ángel Gómez, Joanna Grzymala-Moszczynska, Ali Hamaidia, Qing Han, Mai Helmy, Joevarian Hudiyana, Bertus F. Jeronimus, Ding-Yu Jiang, Veljko Jovanović, Željka Kamenov, Anna Kende, Shian-Ling Keng, Tra Thi Thanh Kieu, Yasin Koc, Kamila Kovyazina, Inna Kozytska, Joshua Krause, Arie W. Kruglanski, Anton Kurapov, Maja Kutlaca, Nóra Anna Lantos, Edward P. Lemay, Cokorda Bagus Jaya Lesmana, Winnifred R. Louis, Adrian Lueders, Najma Iqbal Malik, Anton Martinez, Kira O. McCabe, Jasmina Mehulić, Mirra Noor Milla, Idris Mohammed, Erica Molinario, Manuel Moyano, Hayat Muhammad, Silvana Mula, Hamdi Muluk, Solomiia Myroniuk, Reza Najafi, Boglárka Nyúl, Paul A. O’Keefe, Jose Javier Olivas Osuna, Evgeny N. Osin, Joonha Park, Gennaro Pica, Antonio Pierro, Jonas Rees, Anne Margit Reitsema, Elena Resta, Marika Rullo, Michelle K. Ryan, Adil Samekin, Pekka Santtila, Heyla A. Selim, Michael Vicente Stanton, Samiah Sultana, Robbie M. Sutton, Eleftheria Tseliou, Akira Utsugi, Jolien Anne van Breen, Caspar J. Van Lissa, Kees Van Veen, Michelle R. vanDellen, Alexandra Vázquez, Robin Wollast, Victoria Wai-lan Yeung, Somayeh Zand, Iris Lav Žeželj, Bang Zheng, Andreas Zick, Claudia Zúñiga, N. Pontus Leander

**Affiliations:** 1 Department of Psychology, New York University Abu Dhabi, PO BOX 129188, Saadiyat Island, UAE; 2 University of Virginia, Charlottesville, USA; 3 University of Groningen, Groningen, The Netherlands; 4 University of Amsterdam, Amsterdam, The Netherlands; 5 University of Thessaly, Volos, Greece; 6 International Islamic University Malaysia, Gombak, Malaysia; 7 Pristine University, Pristine, Kosovo; 8 Ankara Science University, Ankara, Turkey; 9 Universidad Peruana de Ciencias Aplicadas, Lima, Peru; 10 University of Sargodha, Sargodha, Pakistan; 11 Sabanci University, Istanbul, Turkey; 12 De La Salle University, Manila, Philippines; 13 Thammasat University, Pathumthani, Thailand; 14 Sungkyunkwan University, Seoul, South Korea; 15 Heriot Watt University, Edinburgh, Scotland; 16 Eötvös Loránd University (ELTE), Budapest, Hungary; 17 University of Belgrade, Belgrade, Serbia; 18 Taras Shevchenko National University of Kyiv, Kiev, Ukraine; 19 Leuphana University Luneburg, Lüneburg, Germany; 20 Sapienza University of Rome, Rome, Italy; 21 University of Kent, Canterbury, UK; 22 Alexandru Ioan Cuza University of Iasi, Iasi, Romania; 23 Duke University, Durham, USA; 24 Universidad Nacional de Educación a Distancia (UNED), Madrid, Spain; 25 Jagiellonian University, Kraków, Poland; 26 University Setif 2, Sétif, Algeria; 27 University of Bristol, Bristol, UK; 28 Menoufia University, Al Minufiyah, Egypt; 29 Universitas Indonesia, Depok, Indonesia; 30 National Chung-Cheng University, Chiayi, Taiwan; 31 University of Novi Sad, Novi Sad, Serbia; 32 University of Zagreb, Zagreb, Croatia; 33 Yale-NUS College, Singapore, Singapore; 34 HCMC University of Education, Ho Chi Minh City, Vietnam; 35 Independent Researcher, Nur-Sultan, Kazakhstan; 36 University of Maryland, College Park, USA; 37 Durham University, Durham, UK; 38 Udayana University, Denpasar, Indonesia; 39 University of Queensland, Brisbane, Australia; 40 Université Clermont-Auvergne, Clermont-Ferrand, France; 41 University of Sheffield, Sheffield, UK; 42 Vanderbilt University, Nashville, USA; 43 Usmanu Danfodiyo University Sokoto, Sokoto, Nigeria; 44 University of Cordoba, Córdoba, Spain; 45 University of Peshawar, Peshawar, Pakistan; 46 Islamic Azad University, Rasht Branch, Rasht, Iran; 47 National Research University Higher School of Economics, Moscow, Russia; 48 NUCB Business School, Nagoya, Japan; 49 University of Camerino, Camerino, Italy; 50 Bielefeld University, Bielefeld, Germany; 51 University of Siena, Siena, Italy; 52 University of Exeter, Exeter, UK; 53 International Islamic Academy of Uzbekistan, Tashkent, Uzbekistan; 54 New York University Shanghai, Shanghai, China; 55 King Saud University, Riyadh, Saudi Arabia; 56 California State University, East Bay, Hayward, USA; 57 Nagoya University, Nagoya, Japan; 58 Leiden University, Leiden, The Netherlands; 59 Utrecht University, Utrecht, The Netherlands; 60 University of Georgia, Athens, Georgia; 61 Lingnan University, Tuen Mun, Hong Kong; 62 Imperial College London, London, UK; 63 Universidad de Chile, Santiago, Chile

**Keywords:** Psychology, Health care, Risk factors

## Abstract

This paper examines whether compliance with COVID-19 mitigation measures is motivated by wanting to save lives or save the economy (or both), and which implications this carries to fight the pandemic. National representative samples were collected from 24 countries (N = 25,435). The main predictors were (1) perceived risk to contract coronavirus, (2) perceived risk to suffer economic losses due to coronavirus, and (3) their interaction effect. Individual and country-level variables were added as covariates in multilevel regression models. We examined compliance with various preventive health behaviors and support for strict containment policies. Results show that perceived economic risk consistently predicted mitigation behavior and policy support—and its effects were positive. Perceived health risk had mixed effects. Only two significant interactions between health and economic risk were identified—both positive.

The SARS-Coronavirus-2 Disease (COVID-19) pandemic is primarily a public health crisis. Preventive health behaviors such as avoiding crowded spaces and social isolating are crucial mitigation measures requested from the population to fight the spread of the COVID-19^1^. However, these mitigation measures rapidly produced unintended effects, generating a collateral economic crisis, in the form of rising unemployment claims, income losses, and a generalized uncertainty about global markets^2,3^. This challenge can be conceptualized as a risk–risk trade-off^4^: actions undertaken to minimize or eliminate certain risks to human health have the perverse effect of promoting others, equally or more problematic than the original risk. This trade-off, occurring on a global scale, is an exceptional feature of this pandemic.

Here, we focus on risk perceptions about the COVID-19. Risk perceptions have proved crucial to understand individuals’ attitudes and behaviors in the face of threat^4^, and how people weigh costs versus benefits when tackling hazards^5^. Research about risk perception is prolific, but mostly focuses on a single, primary hazard causing the threat—e.g., a virus, a hurricane, floods. The dynamics that may occur with secondary or collateral risks has been subjected to less scrutiny. However, this is a crucial point to examine under the current situation. In the COVID-19 pandemic, the primary risk is considered to be contracting the virus, and the economic risk created by the mitigation measures (e.g., unemployment, income loss) is regarded as a secondary risk, which should be tolerated in order to address the primary risk. However, this secondary risk (economic) has taken proportions that rival with the primary risk (health), to the point that some people claim to be against following mitigation measures out of concerns for the economy^6^. Anecdotal evidence and media narratives commonly frame these risks as conflicting forces. But then again, the question about whether economic (vs. health) concerns motivate or discourage following public health measures has not received an empirical answer thus far—notwithstanding the heated debate^1–3,6^.

The goal of this paper is to determine how perceived health risk versus perceived economic risk due to the coronavirus are associated with (a) compliance with preventive health behaviors, including frequent hand washing, avoiding crowded spaces and social isolation, and (b) support for strict containment policies, comprising support for mandatory vaccination, support for mandatory quarantine for those infected or exposed to coronavirus, and reporting suspected COVID-19 cases. This study focuses on individual-level psychological and behavioral processes, although the analysis will control for a variety of macroeconomic and healthcare system variables, previously shown to influence health behavior and health outcomes^7–11^. The analysis involves 24 countries from five continents that cover various levels of economic development and different temporal stages of the COVID-19 pandemic.

There is evidence that the trade-off between the death toll and economic loss is illusory^12^. However, people have often been presented with the binary, mutually exclusive choice—should priority be given to save lives or save the economy?^13^—and preferences tend to favor saving lives, suggesting a higher priority attributed to contain the virus than to boost the economy. The hypothesis deriving from this result would be that risk perceptions about getting infected with the virus should predict how much people comply with protective behaviors and support the containment policies. This is also in line with the concerns expressed about a partisan divide^14^: the virus is being framed with different levels of lethality to distinct political audiences, and these different perceptions about the virus gravity are suggested to influence compliance with mitigation measures.

However, posing the problem as a mutually exclusive choice (lives vs. livelihoods) may not fully capture the complexity of this issue nor provide the most accurate perspective about the intricacies between health and economic risks. Notably, most health mitigation measures need to be followed and sustained in order to safely reopen the economy. This further increases the relevance to understand this association because policy measures impose restrictions and isolation on individuals and households, who are also business owners, employees and consumers.

We will specifically examine whether perceived health and economic risks interact to predict these outcomes. These risks may act synergistically to increase compliance with mitigation measures (positive interaction), or in contrast, these risks may clash, meaning that perceiving a high risk for both health and the economy may lead to conflicting views about mitigation measures (negative interaction). The fact remains that, thus far, it is unclear whether fighting COVID-19 is perceived as a choice between saving lives and saving the economy (or both). Both hypotheses have been raised in national political arenas around the globe. This analysis is critical to inform risk communication strategies that aim to be effective in achieving the public health targets.

This research responds to calls^15^ to understand the psychological factors underlying individuals’ response to this pandemic, mindful that even after the availability of COVID-19 vaccines, behavioral, non-pharmaceutical protective measures remain crucial^16^—but largely dependent on voluntary compliance. Our primary data was collected to examine whether health policy analysis should consider not just governance-level guidelines, but also individual-level decision making as a relevant dimension to understand compliance with policy measures. Policy guidelines may be curtailed if these fail to effectively communicate the relevant risks or, as our data will show, focus its communication on the wrong risks.

## Results

All measures are fully described in Supplementary Table 1. Summary descriptive statistics per country regarding sociodemographic variables, individual and country-level covariates are presented in Supplementary Tables 2 to 5. We start by illustrating the main variables at the individual level with a series of descriptive statistics that control for potential cross-cultural differences in response sets^17,18^ (procedure described in “Materials and methods” section). This descriptive analysis is followed by multilevel regression models that account for individuals nested within countries.

### Global risk perceptions about health and the economy

**Figure 1:**
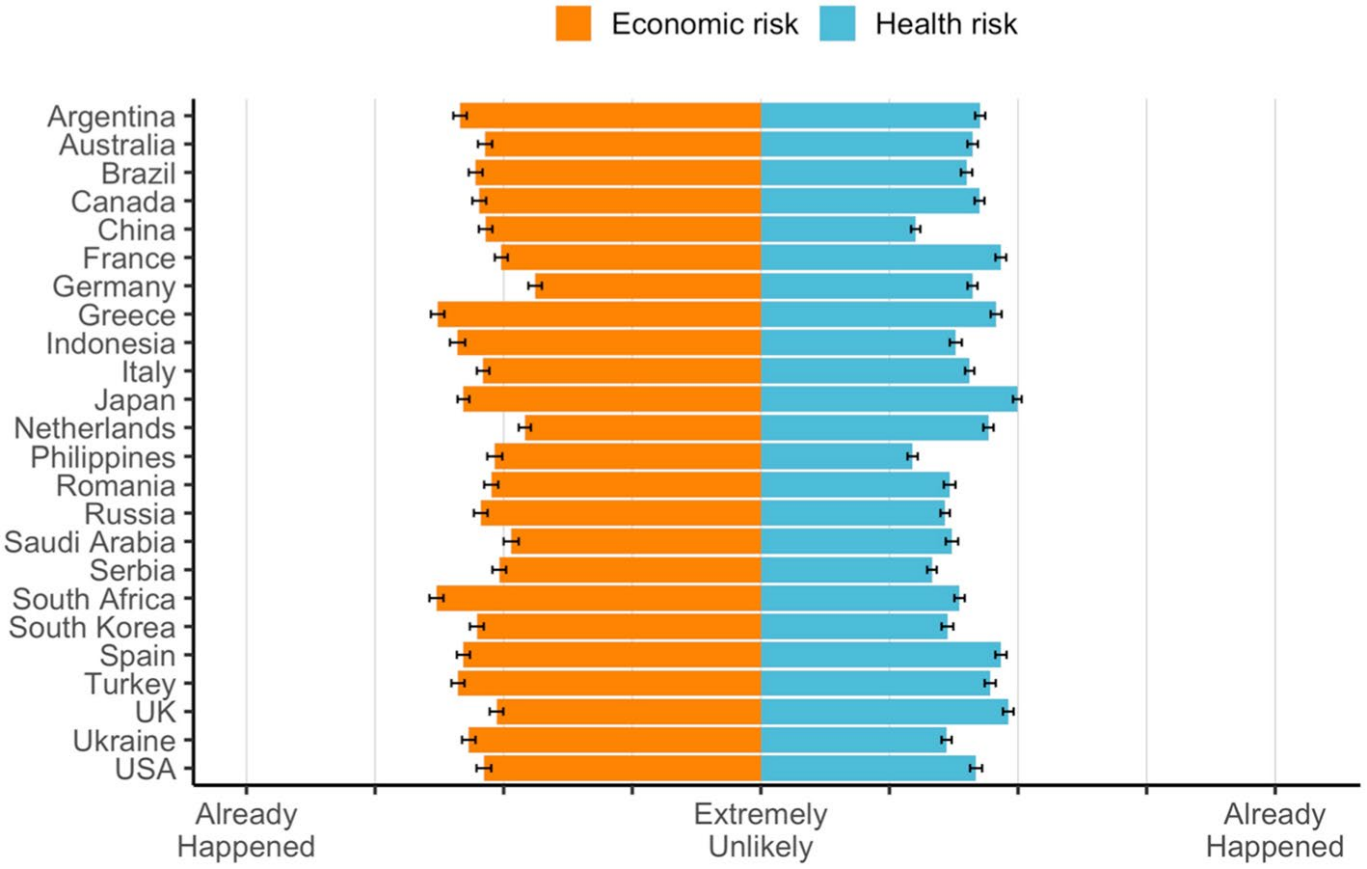
Perceived health risk versus perceived economic risk due to the coronavirus. *Note*: Raw Scores, Error Bars 95% CI. Standardized scores correcting for cross-cultural response sets returned the same country comparative hierarchy per risk. Standardized scores are presented in Supplementary Figs 1 and 2.

Figure 1 presents both the perceived likelihood to get infected with coronavirus and the perceived likelihood to suffer economic losses due to the coronavirus. Globally, average ratings suggest a low perceived risk to get infected with the virus (*M* = 3.23 *SD* = 1.43 95% CI 3.21–3.24; mean significantly below 4 or scale mid-point *t*(25,370) = − 86.19 *p* < .001; median = 3). Regarding economic risk perception, average perceptions suggest a moderate perceived risk (M = 4.35 SD = 1.80 95% CI 4.33–4.37; mean significantly above 4 or scale mid-point *t*(25,382) = 30.91 *p* < .001). Perceived health risk and economic risk are moderately correlated (*r* = .31 *p* < .001) (full country breakdown per risk perception in Supplementary Table 3).

**Figure 2:**
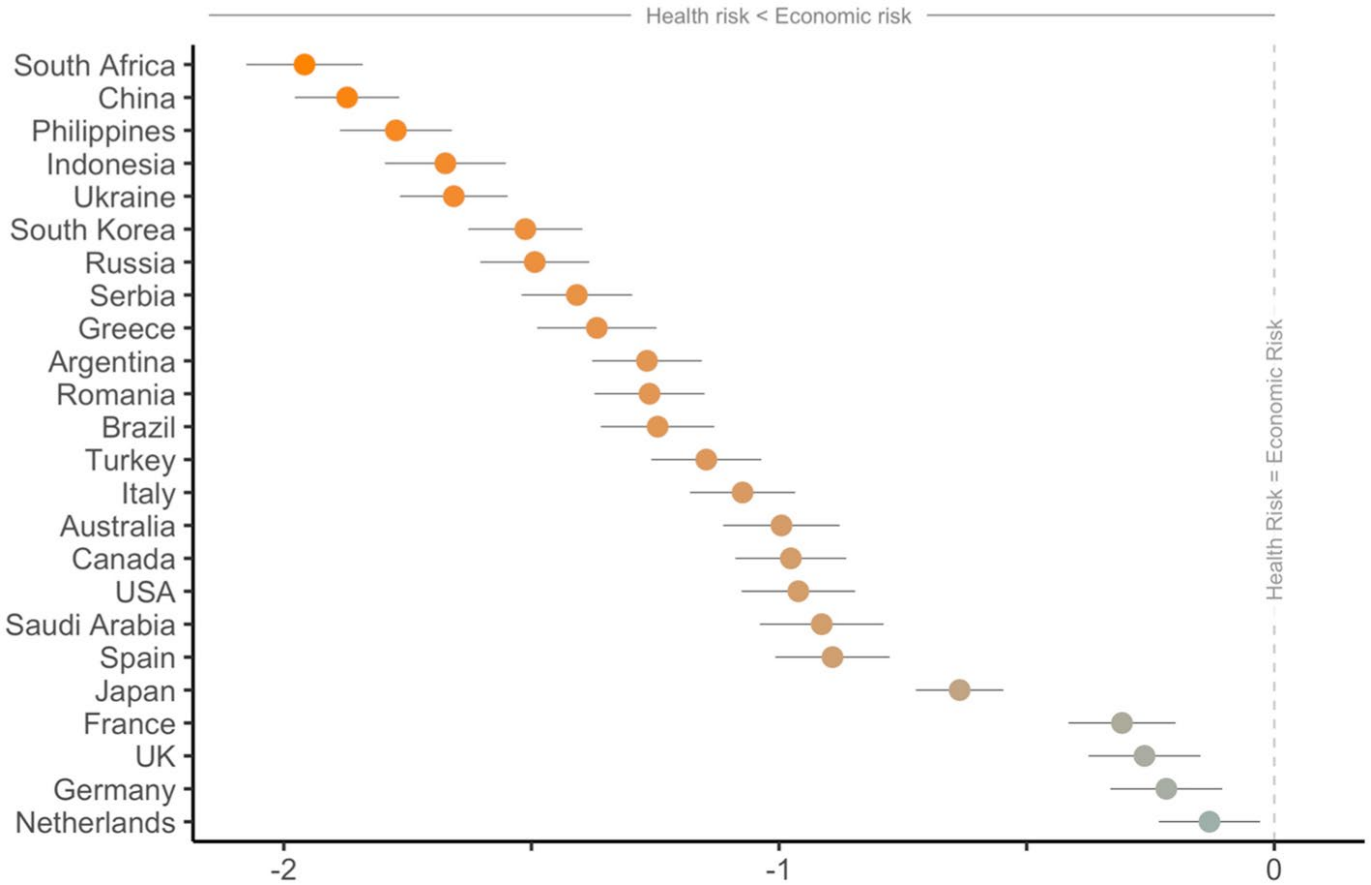
Mean difference between perceived health risk and perceived economic risk. *Note*: Standardized Mean Difference, Error Bars 95% CI.

**Figure 3:**
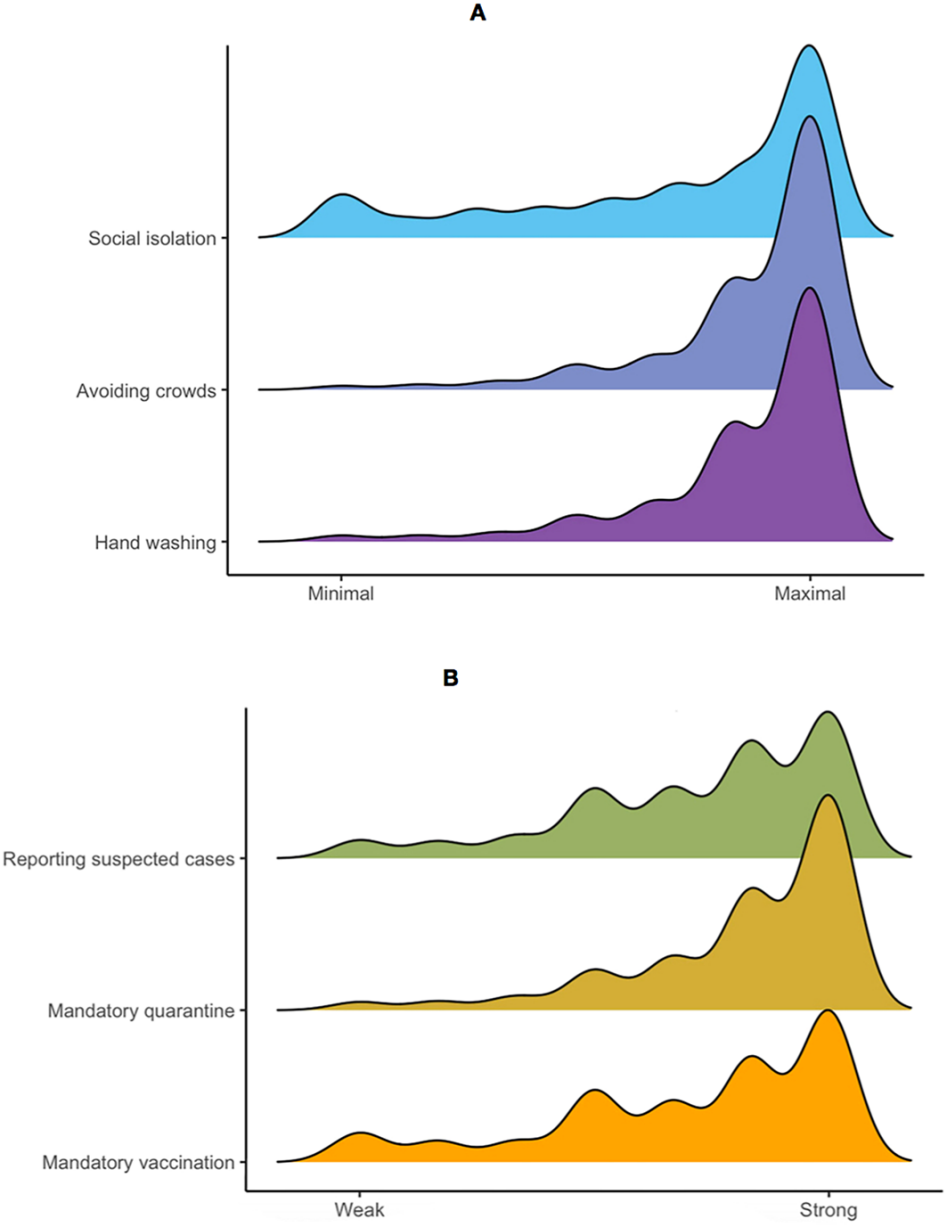
Density plots for compliance with preventive health behaviors (upper figure **A**) and support for containment policies (lower figure **B**).

Figure 2 further shows that people worldwide expect to suffer economically more than in terms of health (all paired samples *t tests p* < .01; M_diff_ = − 1.12 SD = 1.92 95% CI − 1.15, − 1.10; median = − 1). Perceiving a higher risk to suffer economic losses, than to get infected with the virus, is also a pattern consistent across sociodemographic groups. Different population groups regarding age, gender, education, financial and employment status, and political ideology, unanimously report a higher perceived economic risk (vs. health risk) due to the coronavirus (all paired *t* *tests p* < .001). More precisely, perceptions about health and economic risks differ between groups (e.g., people under 25 perceive a lower health risk compared to all other ages), but perceived economic risk is reliably higher in pairwise comparisons within all subgroups (further information about differences per perceived risk within each sociodemographic category are presented in Supplementary Materials—Figure 3).

### Global compliance and support for mitigation measures

We now turn to the analysis of global compliance with preventive health behaviors and support for strict containment policies (country breakdown per outcome is presented in Supplementary Tables 4 and 5). Overall, compliance with crowd avoidance is high (83% agree or strongly agree complying/supporting this measure), followed by frequent hand washing (81%), and to a lower degree, social isolation from family and friends other than household members (55%). Regarding the support for strict containment policies, the most supported measure would be mandatory quarantine for those that have or have been exposed to coronavirus (73%). Both mandatory vaccination for coronavirus (56%), and reporting suspicious COVID-19 cases (57%) would be less approved. Figure 3 shows the density plots for these six outcomes.

### Association between risk perception and mitigation measures

To examine how risk perceptions are associated with these six outcomes, we conducted several multilevel regression models. Given the hierarchical nature of the data, with individuals nested within countries, multilevel regression was used to adjust for the dependence in the data and possible confounders (step-by-step analyses taken, and detailed parameters of the models are fully described in “Materials and methods” section). All models controlled for COVID-19 case-fatality rate per country (total COVID-19 deaths per million/total COVID-19 cases per million). The models also tested a quadratic term for health risk due to exploratory visual analyses suggesting curvilinear relationships between health risk (but not economic risk) and several outcomes (see exploratory plots in Supplementary Figure 4). Moreover, individual and country-level covariates were included in the last step (Model 2), informed by previous research as potential predictors of health behavior and health outcomes^7–11^ (covariates are fully described in “Materials and methods” section, and Supplementary Tables 1 and 2; regression coefficients for covariates per regression model presented in Supplementary Tables 6 and 7). The multilevel regression models predicting preventive health behaviors are displayed in Table 1, while models predicting support for strict containment policies are presented in Table 2.

**Table 1:** Multilevel regression modeling: preventive health behaviors.

	Hand washing			Avoid crowds			Social isolation		
	0	1	2	0	1	2	0	1	2
Intercept	0.02 (.06)	0.02 (0.05)	0.00 (0.03)	− 0.01 (0.06)	0.00 (0.06)	0.01 (0.03)	− 0.04 (0.08)	− 0.08 (0.23)	− 0.04 (0.06)
Control: case-fatality rate	− 0.02 (.04)	− 0.02 (0.04)	0.00 (0.02)	0.01 (0.04)	0.00 (0.04)	− 0.01 (0.02)	0.04 (0.06)	0.11 (0.06)	0.06 (0.04)
Health risk (HR)		0.01 (0.02)	0.03 (0.02)		0.00 (0.02)	0.02 (0.01)		0.01 (0.01)	0.01 (0.01)
Economic risk (ER)		0.11*** (0.01)	.11*** (0.01)		0.09*** (0.01)	0.10*** (0.01)		0.05*** (0.01)	0.06*** (0.01)
HR × ER		0.01 (0.01)	.02** (0.01)		0.00 (0.01)	0.01 (0.01)		− 0.02** (0.01)	− 0.01 (0.01)
Health Risk^2^ (HR^2^)		− 0.01 (0.01)	− 0.01 (0.00)		0.00 (0.01)	0.01 (0.00)		− 0.03*** (0.01)	− 0.02** (0.01)
HR^2^ × ER		0.00 (.00)	− 0.01 (0.00)		0.01 (0.00)	0.00 (0.00)		0.00 (0.00)	0.01 (0.00)
Adjusted ICC	0.04	00.05	0.02	0.04	0.05	0.02	0.09	0.05	0.03

Reporting unstandardized coefficients, standard errors in parentheses. *p < .05, **p < .01, ***p < .001. All predictors are presented in the “Materials and methods" section and detailed in Table S1. All models controlled for COVID-19 case-fatality rate: total COVID-19 deaths per million/total COVID-19 cases per million. Model 2 adjusted for individual and country level covariates as follows. Individual level covariates: (1) direct exposure to someone in their personal network (self, family, friends) infected with COVID-19; (2) perceived knowledge about the COVID-19, (3) perceived knowledge about the economic consequences of the COVID-19; (4) the perceived quality of the public messages received, (5) community norms about mitigation measures, and (6) sociodemographic variables (age, gender, education, employment and financial status). Country-level covariates included (1) total population of the country (in millions), (2) gross domestic product (GDP) per capita (in current $US), (3) unemployment rate estimates for 2020 (as % of the labor force), (4) old age dependency ratio (%), (5) Gini Index, (6) general health expenditure (as %GDP), (7) private health expenditure (as % health expenditure), (8) out-of-pocket health payments (as % health expenditure), (9) number of hospital beds (per 1000 people).

**Table 2:** Multilevel regression modeling: support for strict containment measures.

	Mandatory vaccination			Mandatory quarantine			Report suspected cases		
	0	1	2	0	1	2	0	1	2
Intercept	− 0.09 (0.08)	− 0.05 (0.07)	− 0.04 (0.06)	0.00 (0.07)	0.00 (0.06)	0.01 (0.03)	− 0.04 (0.08)	− 0.08 (0.23)	− 0.04 (0.06)
Control: case-fatality rate	0.09 (0.06)	0.04 (0.04)	0.04 (0.04)	0.00 (0.05)	0.00 (0.04)	− 0.01 (0.02)	0.04 (0.06)	0.11 (0.06)	0.06 (0.04)
Health risk (HR)		0.05*** (0.01)	0.06*** (0.01)		0.00 (0.02)	0.02 (0.01)		0.01 (0.01)	0.01 (0.01)
Economic risk (ER)		0.03* (0.02)	0.04** (0.14)		0.09*** (0.01)	0.10*** (0.01)		0.05*** (0.01)	0.06*** (0.01)
HR × ER		0.01 (0.01)	0.01* (0.01)		0.00 (0.01)	0.01 (0.01)		− 0.02** (.01)	− 0.01 (0.01)
Health Risk^2^ (HR^2^)		0.00 (0.01)	0.00 (0.01)		0.00 (0.01)	0.01 (0.00)		− 0.03*** (0.01)	− 0.02** (0.01)
HR^2^ × ER		0.00 (0.00)	0.00 (0.00)		0.01 (0.00)	0.00 (0.00)		0.00 (0.00)	0.01 (0.00)
Adjusted ICC	0.04	0.08	0.05	0.04	0.05	0.02	0.09	0.005	0.03

Reporting unstandardized coefficients, standard errors in parentheses. *p < .05, **p < .01, ***p < .001. All predictors are presented in “Materials and methods” section and detailed in Table S1. All models controlled for COVID-19 case-fatality rate: total COVID-19 deaths per million/ total COVID-19 cases per million. Model 2 adjusted for individual and country level covariates as follows. Individual level covariates: (1) direct exposure to someone in their personal network (self, family, friends) infected with COVID-19; (2) perceived knowledge about the COVID-19, (3) perceived knowledge about the economic consequences of the COVID-19; (4) the perceived quality of the public messages received, (5) community norms about mitigation measures, and (6) sociodemographic variables (age, gender, education, employment and financial status). Country-level covariates included (1) total population of the country (in millions), (2) gross domestic product (GDP) per capita (in current $US), (3) unemployment rate estimates for 2020 (as % of the labor force), (4) old age dependency ratio (%), (5) Gini Index, (6) general health expenditure (as %GDP), (7) private health expenditure (as % health expenditure), (8) out-of-pocket health payments (as % health expenditure), (9) number of hospital beds (per 1000 people).

Intraclass correlation coefficients (ICC) are very small across all models, particularly when individual and country covariates are introduced in Model 2. The proportion of variance explained by country ranges between 2 and 12%, suggesting the relationship between perceived risks and following mitigation measures is mostly explained by individual differences across countries.

Results show that perceived economic risk consistently predicts following the COVID-19 mitigation measures, and that this relationship is linear. The more people perceive themselves to be at risk of suffering economic losses due to the coronavirus, the more people comply with all preventive health behaviors and support strict compliance policies. Alternatively, individuals' perceived risk of getting infected with the coronavirus had no association with the two most followed preventive health behaviors: frequent hand washing (linear *B* = .03 *p* = .11; quadratic *B* = − .01 *p* = .07) and avoiding crowded spaces (linear *B* = .02 *p* = .06; quadratic *B* = − .01 *p* = .13). Positive linear associations for health risk were identified with support for two strict compliance measures: the more people perceived a personal health risk, the more they support mandatory vaccination (*B* = .06 *p* < .001) and support mandatory quarantine (*B* = .03 *p* = .02). Moreover, curvilinear relationships with health risk were also found. Social isolation has a negative quadratic association with health risk (*B* = − .02 *p* < .001). This suggests people increasingly comply with social isolation up to when their perceived infection risk increases to a moderate level, but this compliance decreases when health risk is perceived to be very high. In contrast, health risk has a positive quadratic association with support for reporting suspect cases (*B* = .02 *p* < .001). This implies that people show reduced support for reporting possible COVID-19 cases as their own personal risk increases, but only up to the point of moderate risk. For high levels of perceived health risk, people are more supportive of reporting suspect cases. Although the curvilinear patterns are idiosyncratic, altogether they illustrate that increases in perceived health risk are not a reliable predictor of compliance with mitigation measures.

**Figure 4:**
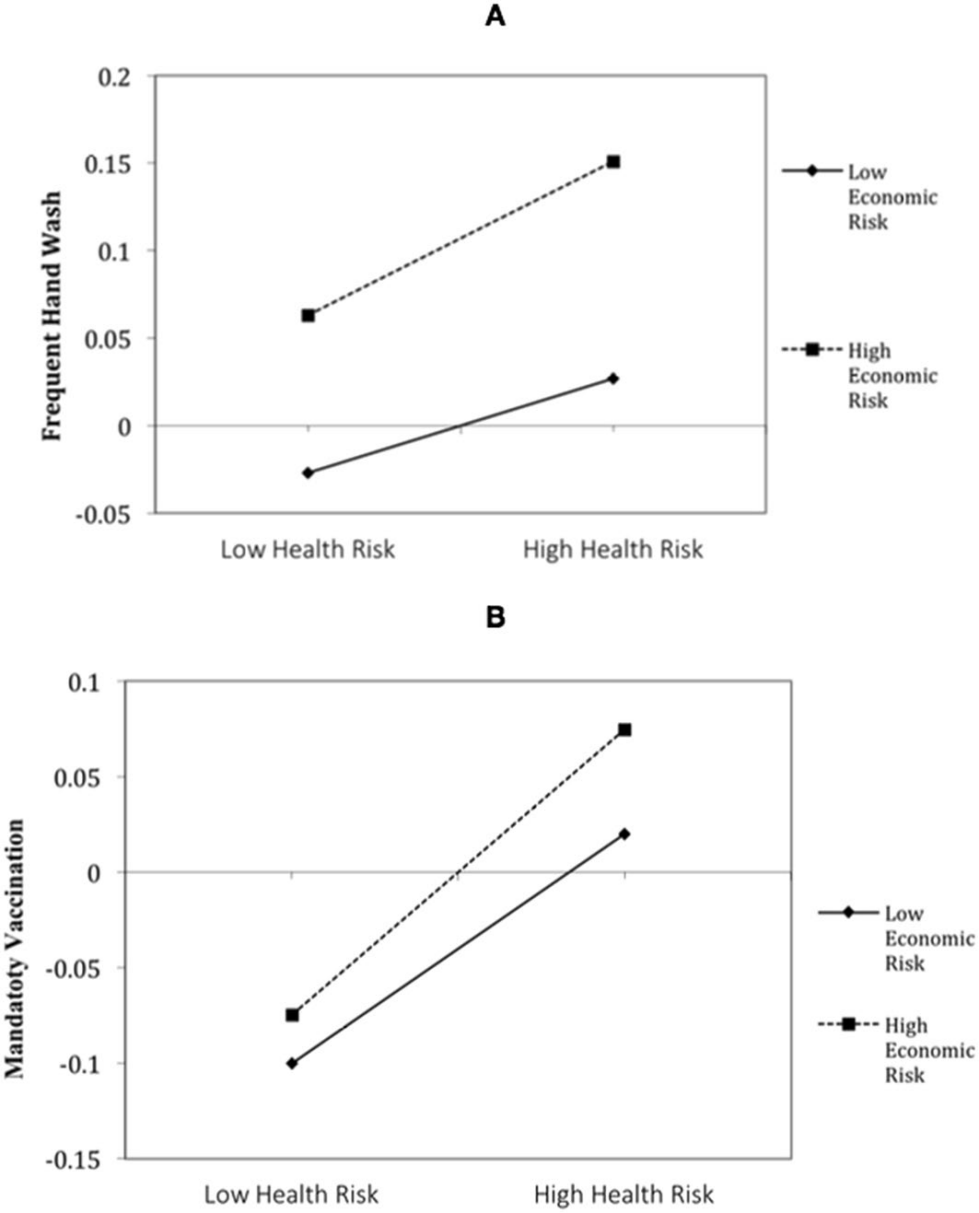
Positive interaction between health and economic perceived risks in their association with frequent hand wash (upper figure **A**) and support for mandatory vaccination (lower figure **B**).

Interaction effects were significant in two out of six cases, both positive for frequent hand washing (*B* = .02 *p* < .001) and support for mandatory vaccination (*B* = .01 *p* = .02). Figure 4 plots these positive interactions. Without controlling for individual and country covariates, the interaction between these risks is negative for social isolation (*B* = − .02 *p* < .01), but no longer reaches significance in Model 2 (*p* = .07).

We conducted a sensitivity analysis to examine the extent to which the results would hold in the subgroup with the lowest economic risk perception: people with no or low financial insecurity. It could be argued that the global pattern of results reflected a generalized modest financial situation by the participants. Nonetheless, results restricted to people who perceive to be financially comfortable largely corroborate the global results (Supplementary Table 8). In this subgroup, the three most accepted measures were also positively predicted by economic risk (hand washing *B* = .06 *p* = .05; avoid crowded spaces *B* = .06 *p* < .01; mandatory quarantine *B* = .07 *p* < .001). This implies that, although people are financially secure, increases in their perceived economic liability are associated with following these measures more. Similar results were also found for mandatory vaccination, predicted by health risk as in the global results (*B* = .06 *p* < .001). Differences were found in the two instances that exhibit more complex relationships between risk perception and behavior: social isolation and reporting suspect cases. Social isolation was only predicted by health risk (*B* = .04 *p* < .01) and risk perception was not associated with support for reporting suspect COVID-19 cases. No interactions nor quadratic effects were found in this subgroup, suggesting that financial security simplifies individuals’ psychological rapport with COVID-19 mitigation measures.

Lastly, we have also conducted sensitivity analyses trying a specification with country-level fixed-effects, and a LASSO specification run on Model 2. The results remain largely unchanged in these two additional specifications, and are presented in Supplementary Tables 9 and 10.

## Discussion

This work sheds an empirical light into one the most heated debates in the era of COVID-19. We examined global risk perceptions regarding contracting the virus and suffering economic losses due to the pandemic, and both their association with compliance and support for the mitigation measures to fight COVID-19. The key takeaway is that, globally, people are not perceiving saving lives and saving the economy as dueling goals. This work suggests that that the path forward should not be to ignore the virus nor minimize its dangers to reopen the economy, nor to focus on health vulnerabilities and lives lost to increase preventive health behaviors. Inversely, public messaging may be more effective if delivering the message that COVID-19 mitigation measures need to be followed to avoid (further) economic and job losses. This key takeaway derives from a number of important results uncovered in this work.

First, on average, global risk perceptions are low to moderate. Despite the widespread disarray created by the coronavirus, relentless media coverage, and the high volume of cases and death toll, people perceive contracting the virus as an unlikely event. Across all countries examined, the highest perceived likelihood to get infected with coronavirus only reached a fifty-fifty chance. Perceived as a more likely prospect is the risk of suffering economic consequences due to the coronavirus. Average economic risk perceptions are moderate: people think that experiencing economic losses is somewhat likely. The higher perceived economic risk (vs. the health risk) from the coronavirus is a remarkably consistent pattern across all countries and social groups regardless of age, gender, education, employment and financial status and political ideology. These results suggest that risk perceptions seem to accurately reflect the objective probabilities reported by international organizations regarding both risks. The probability to get infected with the virus is considered to be low to moderate for the general population^19^, whereas the probability to suffer economic losses is nearly 50% for the global workforce^20^. Therefore, at the aggregate level, people seem to be correctly assessing their relative vulnerability regarding these risks.

Second, perceived economic risk—and not health risk—is the main predictor of mitigation behavior and policy support. Moreover, its effects are positive. According to our data, only economic concerns positively predicted all outcomes. This association is unrelated to the fact that economic risk is perceived to be higher; instead, it indicates that it is the variation in perceived economic risk that is co-varying with changes in compliance and support for COVID-19 measures. The more people perceive a personal risk to suffer economic losses due to the pandemic, the more they frequently wash their hands, avoid crowds, socially isolate, support mandatory vaccination, mandatory quarantine for those that have coronavirus or who have been exposed to the virus, and support reporting suspected COVID-19 cases. Based on these results, the view^6^ that some people seem to be against following mitigation measures because of their concerns about the economy is not supported as a mainstream perspective.

Perceived health risk exhibited mixed effects. The strongest associations with health risk were support for mandatory vaccination and mandatory quarantine. Null effects were found for the two most followed preventive health behaviors: frequent hand washing and avoiding crowded spaces. Furthermore, results also showed quadratic effects of health risk on support for the strictest measures such as social isolation and reporting suspect COVID-19 cases. Regarding social isolation, if people perceive contracting the virus as very unlikely, the sacrifice to socially isolate may not seem worth it. If personal virus infection risk increases too much, people don’t want to be isolated from friends and family, possibly as a coping mechanism against rising anxiety and fear. Regarding the support for reporting suspect cases, results imply that the burden of reporting suspected COVID-19 cases would only be undertaken when people perceive themselves either at a very low or very high health risk. That is, they would only support such a measure when they think it could not happen to them, or when the fear of infection is so high that it justifies drastic action. There is a precedent for people having conflicted psychological attitudes towards restrictive policies, often more supported when it mostly affects others, but assessed negatively when it affects themselves^21^. This suggests that while strict policies are expected to better contain the virus spread, more moderate measures may have higher public acceptability and less behavioral backlash.

Third, few significant interactions between health risk and economic risk were identified, and when found, these were positive interactions. These risks do not appear to work as competing forces, but mostly as independent main effects that positively contribute towards mitigation behavior—with a stronger contribution from economic risk. In the case of the positive interactions identified, health and economic risk collaborate to increase frequent hand washing and supporting mandatory vaccination. We interpret this positive interaction as a sign that neither of these measures affect economic activities, and both protect personal and public health. Our data did not include willingness to wear face masks in public nor compliance with public social distancing, although our results suggest that these could also be instances of a positive interaction between health and economic risks. Both face masks and keeping a distance from others in public spaces protect health while preserving the continuity of economic activities. No significant negative interactions were identified, which could have been expected for measures that protect health at the cost of reduced economic interactions i.e., mandatory quarantine and social isolation. Therefore, overall, this paper does not suggest corroboration for the narrative that regular people engage in the health versus economy zero-sum thinking, often disseminated in journalistic, political and business messaging.

Last, there were null effects from case-fatality rates, included in all models as a control variable. The number of COVID-19 deaths and cases, and their ratio (case-fatality rate), are some of the most frequently publicized pieces of information about the pandemic, yet seemingly unrelated to following protective health behaviors and supporting containment measures, with or without controlling for covariate factors. This may suggest the need to shift public health messaging away from COVID-19 health statistics, and more towards economic statistics.

In conclusion, we show that economic concern is a better predictor of virus prevention behavior and support for strict health policies to contain the virus, compared to the concern about getting infected with coronavirus. In other words, some people may deny the seriousness of the virus^14^ but fewer are denying the economy is being affected. Hence, a focus on economic threats is universally shared and can be a way to unify people around a common goal. This raises the question of whether appealing to personal economic risk is a more effective way to motivate virus mitigation behavior, rather than appealing to personal virus (health) risk.

Nonetheless, some limitations in this work should be addressed in future research. An important point is that no causality can be attributed to risk perception in its effects on mitigation behavior and policy support. Cross-sectional designs are liable to the possibility of reverse causality, by which it would be following mitigation measures that decreases perceived (and objective) risk. Although this is an open possibility, we argue that it is unlikely that frequent hand or avoiding crowds would reduce perceived economic risk, but not health risk. Furthermore, the logic of reverse causality would only apply to personal behaviors reducing personal risk, but less so to how more positive attitudes towards potential containment policies decrease perceived risk. We maintain that our version of causality is more parsimonious across all outcomes. Nonetheless, other research designs (e.g., longitudinal studies, quasi-experimental designs examining survey data in individuals affected by different lockdown measures) are needed to establish the direction of this relationship more conclusively^22^.

Another noteworthy point is that, given the large sample sizes involved, effects small in magnitude were statistically significant results. This applies both to main effects and interaction effects. Therefore, even though economic risk seems to be a better predictor of compliance and support for mitigation measures, compared to health risk, both these factors offer a low contribution to understand what drives people to follow COVID-19 measures. Nonetheless, small effects can add up to substantial effects when scaled-up to the population level^23^. For example, even though smoking is one of the greatest behavioral risk factors for developing lung cancer or heart disease, the 10-year absolute risk for a heavy smoker to develop lung cancer is only 0.3% and the risk of developing heart disease is only 0.9%^24^. And yet, these small effects have tremendous significance from a population perspective, with hundreds of thousands of heavy smokers dying prematurely. Given that the COVID-19 pandemic literally has a global reach, small effects matter. Therefore, risk communication strategies that potentially influence risk perceptions about personal risk may add up to a substantial increase in compliance and support for mitigation measures.

A concluding remark is that future research should explore further the role of country/ culture characteristics^17,18^ in modulating individual perceptions about the health and economic risks posed by the COVID-19. Countries differ in the characteristics of their healthcare (e.g., no access to free healthcare) and economic systems (e.g., high unemployment rate), and in their overall organizational capacity to buffer the population from this challenge. Our analysis did not dwell upon this subject, although our results from ICC and country-level covariate analysis suggest that country differences play a small role. Nonetheless, a more in-depth cross-country analysis may uncover the need for a cultural adjustment to risk communication.

## Materials and methods

### Study design and data collection

This cross-sectional study is part of the global Psycorona project (https://psycorona.org) which focuses on how people feel and think about the coronavirus epidemic and its economic consequences. This study complied with ethical regulations for research on human subjects and all participants gave informed consent, as approved by the Institutional Review Board at New York University Abu Dhabi (protocol HRPP-2020-42) and the Ethics Committee of Psychology at Groningen University (protocol PSY-1920-S-0390). Personal identifiers were removed from all sections of the manuscript, including supplementary information and public dataset.

Survey responses were collected through Qualtrics’ panel management service, except in China where data was collected by WJX, following a similar methodological approach. The company's methodology involves obtaining responses from invited internet users drawn from its panel of over 90 million people worldwide. Data was collected in 24 countries: Argentina, Australia, Brazil, Canada, China, France, Germany, Greece, Indonesia, Italy, Japan, Netherlands, Philippines, Romania, Russia, Saudi Arabia, Serbia, South Africa, South Korea, Spain, Turkey, United Kingdom, Ukraine, and the United States of America. These countries cover various levels of economic development as well as different temporal stages of the COVID-19 pandemic. National proportional quota samples were collected with a 3% margin of error and 95% confidence level, representative of the country’s population in terms of gender and age (age representativeness was less achieved in China, Greece, Saudi Arabia, Indonesia and the Philippines, where people aged 55+ were less present in the survey). Data quality control was conducted by (1) examining IP addresses to detect potential duplicate responders; and (2) removing participants from the database whose answers indicated random responses. Data was collected online between 10th April and May 11th 2020.

### Measures

All measures are fully described in the Supplementary Table 1. The main predictors were the perceived likelihood to get infected with coronavirus, the perceived likelihood to suffer economic consequences due to the coronavirus, and their interaction effect. A total of six outcomes were predicted. The primary outcomes were compliance with preventive health behaviors, including frequent hand washing, avoiding crowded spaces and social isolation (i.e., no contact with friends and family other than household members). The secondary outcomes were the support for strict health measures, namely support for mandatory coronavirus vaccination, mandatory quarantine for those that have coronavirus and those that have been exposed to the virus, and reporting of suspected coronavirus cases. We chose to examine these items individually, as informative in their own right. However, single item measures do not allow for an internal consistency analysis. Nonetheless, a reliability analysis of the six items (outcomes)—as a measure of overall acceptability of public health measures—reveals a good internal consistency (α = .77).

Several individual and country-level predictors—previously shown to be associated with preventive health behavior and health outcomes^7–11^—were added as covariates in multilevel regression models. Individual-level covariates were (1) direct exposure to someone (self, family, friends) in their personal network infected with COVID-19; (2) knowledge about the COVID-19, (3) knowledge about the economic consequences of the COVID-19; (4) the quality of the public messages received, (5) community norms about mitigation measures, and (6) sociodemographic variables (age, gender, education, employment and financial status, and political ideology). As part of a larger research project PsyCorona, there were other psychological measures collected that were ultimately not selected as covariates due to low theoretical justification. The full set of psychological measures collected can be found here https://psycorona.org/about/. Country-level covariates included (1) total population of the country (in millions), (2) gross domestic product (GDP) per capita (in current $US), (3) unemployment rate (as % of the labor force), (4) old age dependency ratio (%), (5) Gini Index, (6) general health expenditure (as %GDP), (7) private health expenditure (as % health expenditure), (8) out-of-pocket health payments (as % health expenditure), (9) number of hospital beds (per 1000 people).

### Sample

Summary statistics for each country regarding sociodemographic variables and individual and country level covariates are presented in the Supplementary Table 2. At the aggregate level, the sample was gender balanced (51% women), with 52% up to 44 years of age, and 48% aged 45 to old age (range 18–85). Most participants were educated up to completed high school (59%), and the remaining with a completed higher education (19% with Bachelor degree and 13% with postgraduate studies). Most participants were employed (57%, either part- or full-time), and about a third (35%) reported difficulties paying for their expenses. Politically, 40% self-categorize as left leaning, whereas 50% self-categorize as right leaning (about 10% other/no political preference). The analysis includes participants who have already contracted the virus (n = 142) and/ or who have already lost their jobs (n = 1295).

### Statistical analysis

To the best of our knowledge, previous literature about multiple risks or risk interaction was slim to confidently propose or guide in hypotheses formulation. Thus, we opted for not formalizing nor pre-registering any hypotheses^25^. We conducted exploratory analyses examining the relative association between health and economic risks and multiple outcomes related to following mitigation measures. This analysis controlled for several covariates at the individual and country level, theoretically justified^7–11^.

Descriptive differences between countries and between risk perceptions were examined with analysis of variance (ANOVA), LSD and Tukey HSD post hoc tests, and paired samples *t tests.* We classified correlations (*r*) and betas as small if between 0.05 and 0.19, moderate between 0.20 and 0.49, and large if above 0.50, as characteristic in the social sciences^26^.

Different response sets between countries were controlled for by standardizing health and economic risk for the cross-country comparisons in the descriptive statistics. Raw scores on risk perception and the six outcomes were averaged to create a within-subject response average. This average was then subtracted from the raw scores of perceived health risk and perceived economic risk to generate standardized scores for these two variables^17,18^. Given that this procedure did not change the average results and country comparisons, we presented the raw score for a better interpretability by the reader. We, nevertheless, present the standardized health and economic risk standard scores per country in Supplementary Figures 1 and 2.

We estimated the Intraclass Correlation Coefficient (ICC) to describe the correlation among observations within the countries. The ICC is also equivalent to the variance partition coefficient, which can be interpreted as the proportion of variation that is due to a variation between countries^27^.

We also applied hierarchical models^28^ to understand the effects of controlling for person-level predictors taking into account the random variations across nations. In preparation to run these models, we eliminated the missing values from the entire dataset (n = 592), considering all the variables together. If a subject had missing values, all variables from the subject were eliminated. We detected the multivariate outliers using Mahalanobis’ distance and chi-square distribution (ɑ = .95) with a total of 2282 eliminated. The total sample used in the models was N = 22,561, constant across models. The predictors from the individual-level were group-mean-centering by country (and scaling is done by dividing the (centered) columns of x by their standard deviations). Country-level variables used grand-mean-centering, given these have a single value for each country. The models were implemented using R and the package lme4^29,30^. To predict each of the six outcomes, a total of three nested models were selected from a range of 15 models (using ANOVA approach for between model comparison).

The selected models vary in increasing complexity. All models controlled for COVID-19 case-fatality rate: total COVID-19 deaths per million/ total COVID-19 cases per million. Model 0: Model 0 or empty model provided unadjusted rates for the behavior response (outcome) that accounted for clustering. Model 1: included the individual-level variables perception of risk of infection and economic loss (and their interaction), a quadratic term for health risk (and the interaction with economic risk) as predictors for fixed effects and perception of risk of infection and economic loss as random intercept within the country. The use of the random statement measures the variance in the effects of risk of infection and economic loss on behavior responses across countries. Interaction was not used as a random effect because it led to a non-convergence. Model 2: Same as model one plus the individual-level and country-level covariates described in the section Measures above. Model 2 added these covariates as fixed effects. Political ideology was not included in the multilevel regression analysis due to the high number of missing values in most countries and a complete absence of replies in China. This decision was due to wanting to keep the sample size across all models (N = 22,561). Nonetheless, regression models including political ideology were conducted as a sensitivity analysis and results held across the models.

As we used linear mixed models, the variables were checked for normal distribution, scaled in relation to the mean, and extreme outliers were excluded. Models used the Nelder-Mead optimization algorithm for derivative-free optimization. All reported *p* values are two-sided.

### Ethics approval

This study complied with ethical regulations for research on human subjects and all participants gave informed consent, as approved by the Institutional Review Board at New York University Abu Dhabi (protocol HRPP-2020-42) and the Ethics Committee of Psychology at Groningen University (protocol PSY-1920-S-0390).

### Data re-use disclosure

Some of the variables examined in this paper have been previously used and published in other papers developed by the Psycorona Collaboration. Previous papers, however, examined unrelated research questions. For instance, compliance items have been previously reported in unrelated tests of age and country main effects^31,32^. The infection risk perception item was previously reported in effects on subjective wellbeing^33^.

## Supplementary Information

Supplementary Information 1.

Supplementary Information 2.

Supplementary Information 3.

## Data Availability

The dataset and an example of code for R *lme4* are publicly available at the Open Science Framework https://osf.io/xvyna/.
