# Supplementary material for: Lives versus Livelihoods? Perceived economic risk has a stronger association with support for COVID-19 preventive measures than perceived health risk
Source: Sci Rep. 2021 May 6;11:9669. doi: 10.1038/s41598-021-88314-4 (PMC8102566; doi:10.1038/s41598-021-88314-4)
Supplement: Supplementary file 2 — Supplementary Information 2. [file 41598_2021_88314_MOESM2_ESM.pdf]

Linear mixed model fit by REML. t-tests use Satterthwaite's method ['lmerModLmerTest']
Formula: Hand_wash ~ 1 + control + Health_Risk + Economic_Risk + Health_Risk *
  Economic_Risk + Exposure_COVID + Know_COVID19 + Know_Economic +
  Quality_Messages + Community_Norms + Age + Gender + Education +
  Employment + Financial_Insecurity + COUNTRYTot_Population +
  COUNTRYGDPpc + COUNTRYUnemployment + COUNTRYGini +
COUNTRYOld_age_dependency +
  COUNTRYLife_expectancy + COUNTRYHealth_expenditure +
COUNTRYPrivate_health_insurance +
  COUNTRYOut_of_pocket + COUNTRYHospital_beds + (1 + Health_Risk + Economic_Risk |
Country)
  Data: Model_no_group
Control: lmerControl(optimizer = "Nelder_Mead", optCtrl = list(maxfun = 40000))

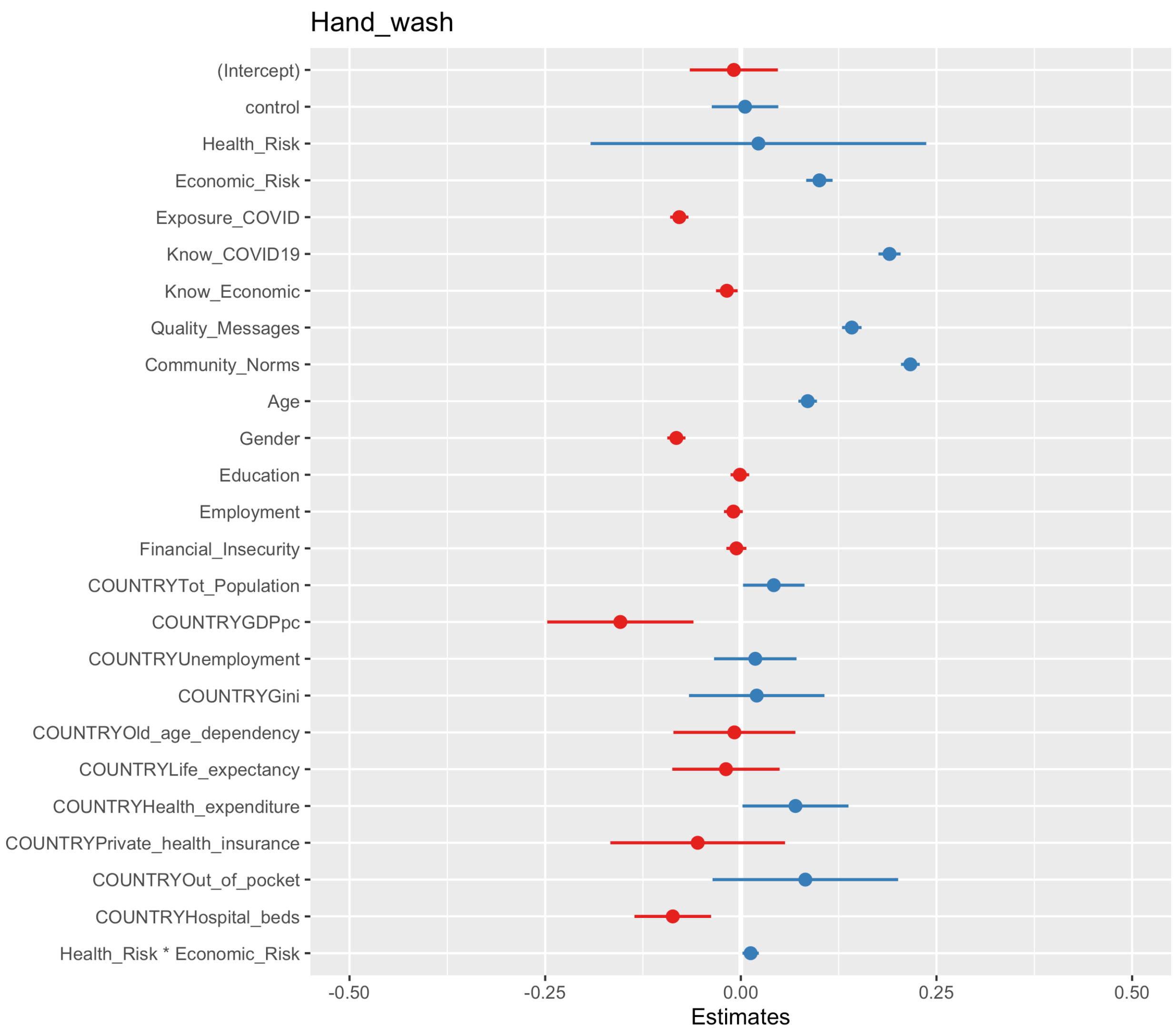

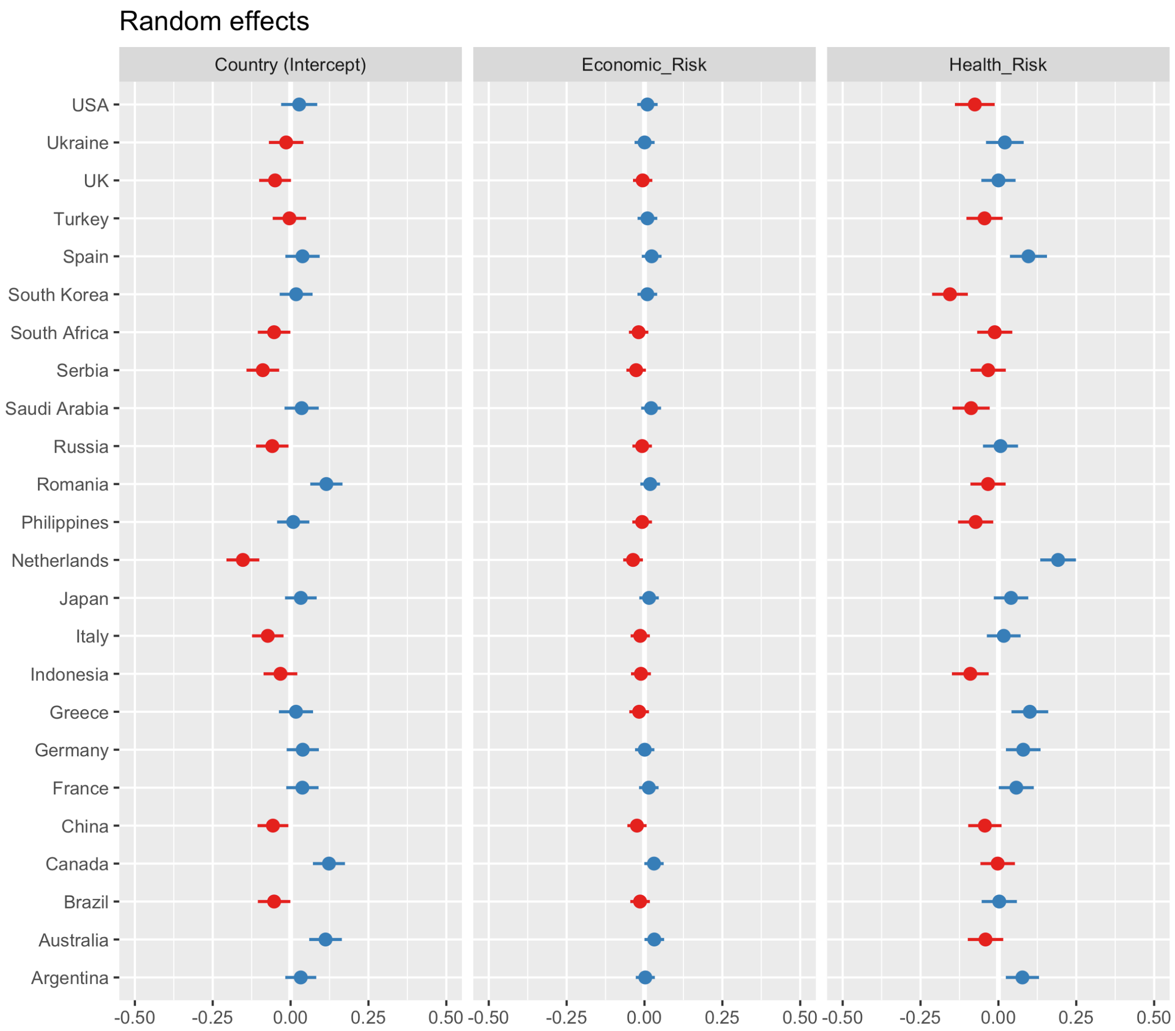
Random effects
Country (Intercept)
Economic_Risk
Health_Risk
USA
Ukraine
UK
Turkey
Spain
South Korea
South Africa
Serbia
Saudi Arabia
Russia
Romania
Philippines
Netherlands
Japan
Italy
Indonesia
Greece
Germany
France
China
Canada
Brazil
Australia
Argentina
-0.50
-0.25
0.00
0.25
0.50
