# Supplementary material for: Lives versus Livelihoods? Perceived economic risk has a stronger association with support for COVID-19 preventive measures than perceived health risk
Source: Sci Rep. 2021 May 6;11:9669. doi: 10.1038/s41598-021-88314-4 (PMC8102566; doi:10.1038/s41598-021-88314-4)
Supplement: Supplementary file 3 — Supplementary Information 3. [file 41598_2021_88314_MOESM3_ESM.pdf]

Supplementary Materials

Lives versus livelihoods? Perceived economic risk has a stronger association with COVID-19 protective measures than perceived health risk

Claudia F. Nisa[1*], Jocelyn J. Bélanger[1], Daiane G. Faller[1], Nicholas R. Buttrick[2], Jochen O. Mierau[3], Maura Austin[2], Birga M. Schumpe[4], Edyta M. Sasin[1], Maximilian Agostini[3], Ben Gützkow[3], Jannis Kreienkamp[3], PsyCorona Collaboration, N. P. Leander[3]

[1] New York University Abu Dhabi, Department of Psychology, UAE
[2] University of Virginia, Department of Psychology, USA
[3] University of Groningen, The Netherlands
[4] University of Amsterdam, The Netherlands
Names and Affiliations detailed in the paper for Psycorona Collaboration

* Corresponding Author: cfn1@nyu.edu. New York University Abu Dhabi, Department of Psychology, Saadiyat Island, PO BOX 129188, UAE

Supplementary Tables

Table S1. Description of variables included, scales used and sources --------------------- 2
Table S2. Sample Description -- 5
Table S3. Summary statistics for risk perceptions --- 9
Table S4. Summary statistics for primary and secondary outcomes (means) ------------- 13
Table S5. Summary statistics for primary and secondary outcomes (%) ------------------ 14
Table S6. Multilevel Regression Modeling: Preventive Health Behaviors ---------------- 16
Table S7. Multilevel Regression Modeling: Support for Strict Containment Measures -- 17
Table S8. Sensitivity Analysis -- 18
Table S9. Fixed-effects Model -- 19
Table S10. LASSO Model --- 20

Supplementary Figures

Figure S1. Standardized health perceived risk per country ------------------------------------ 10
Figure S2. Standardized economic perceived risk per country -------------------------------- 10
Figure S3. Perceived health and economic risk per sociodemographic groups ------------- 11
Figure S4. Association between health and economic risk, and each of the six outcomes - 15

Table S1. Description of variables included, scales used and sources

Variable	Description	Source
**Main Predictors**		
Likelihood to get coronavirus	How likely is it that in the next few months you will get infected with coronavirus [*1- Exceptionally unlikely; 2- Very Unlikely; 3- Unlikely; 4- About equally likely; 5- Likely; 6- Very likely; 7- All but certain; 8=Already happened*]	Survey
Likelihood to suffer economic losses	How likely is it that in the next few months your personal situation will get worse due to economic consequences of coronavirus [*1- Exceptionally unlikely; 2- Very Unlikely; 3- Unlikely; 4- About equally likely; 5- Likely; 6- Very likely; 7- All but certain; 8=Already happened*]	Survey
Interaction Effect	Likelihood to get coronavirus* Likelihood to suffer economic losses	Computed from survey
**Outcome variables**		
Health behavior: Wash hands	To minimize my chances of getting coronavirus, I wash my hands more often *[-3 Strongly disagree; -2 Disagree; -1 Somewhat disagree; 0 Neither agree nor disagree; 1 Somewhat agree; 2 Agree; 3 Strongly agree*]	Survey
Health behavior: Avoid Crowds	To minimize my chances of getting coronavirus, I avoid crowded spaces *[-3 Strongly disagree; -2 Disagree; -1 Somewhat disagree; 0 Neither agree nor disagree; 1 Somewhat agree; 2 Agree; 3 Strongly agree*]	Survey
Health behavior: Social Isolation	In the past 7 days, how much face-to-face social contact have you had with people who live outside your household, namely friends or relatives, in the past 7 days? *[from 0 days to 7 days]* Note: Items reversed to represent isolation (i.e., number of days per week in social isolation).	Survey
Support for Mandatory Vaccination	I would sign a petition that supports mandatory vaccination once a vaccine has been developed for coronavirus *[-3 Strongly disagree; -2 Disagree; -1 Somewhat disagree; 0 Neither agree nor disagree; 1 Somewhat agree; 2 Agree; 3 Strongly agree]*	Survey
Support for Mandatory Quarantine	I would sign a petition that supports mandatory quarantine for those that have coronavirus and those that have been exposed to the virus *[-3 Strongly disagree; -2 Disagree; -1 Somewhat disagree; 0 Neither agree nor disagree; 1 Somewhat agree; 2 Agree; 3 Strongly agree]*	Survey
Support for Suspected Case Report	I would sign a petition that supports reporting people who are suspected to have coronavirus *[-3 Strongly disagree; -2 Disagree; -1 Somewhat disagree; 0 Neither agree nor disagree; 1 Somewhat agree; 2 Agree; 3 Strongly agree]*	Survey
**Individual Level Covariates**		
Gender	1-Female; 2-Male	Survey

Age	Categories 1- 18 to 24; 2 – 25 to 34; 3- 35 to 44; 4- 45-54; 5- 55-64; 6-65+	Survey
Education	Categories 1- Primary education; 2- General secondary education; 3- Vocational education; 4- Higher education; 5- Bachelor's degree; 6- Master's degree; 7- PhD degree	Survey
Political Ideology	1- Left side of the political spectrum; 2=Right side of the political spectrum	Survey
Employment	1- Employed (part-time or full-time); 0-Not employed	Survey
Financial Insecurity	Due to my financial situation, I have difficulties paying for my expenses *[-2 Strongly disagree; -1 Disagree; 0 Neither agree nor disagree; 1 Agree; 2 Strongly agree]*	Survey
Exposure COVID-19 personal network	Do you know someone who has been infected with coronavirus? Possible answers: (i) a family member, (ii) a close friend, (iii) someone else [*answer yes/no: sum of answers reported in database*]	Survey
Knowledge COVID-19	How knowledgeable are you about the recent outbreak of CoVID-19, commonly referred to as the Coronavirus? *[1- Not at all knowledgeable; 2-Slightly knowledgeable; 3-Moderately knowledgeable; 4-Very knowledgeable; 5-Extremely knowledgeable]*	Survey
Knowledge Economic consequences	How knowledgeable are you about the potential economic and financial consequences of coronavirus? *[1- Not at all knowledgeable; 2-Slightly knowledgeable; 3-Moderately knowledgeable; 4-Very knowledgeable; 5-Extremely knowledgeable]*	Survey
Quality Message	To what extent are you getting clear, unambiguous messages about what to do about the Coronavirus? [*1- Messages are completely unclear/ambiguous to 6- Messages are very clear/unambiguous*]	Survey
Community Norms	Right now, people in my area do self-isolate and engage in social distancing *[-3 Strongly disagree; -2 Disagree; -1 Somewhat disagree; 0 Neither agree nor disagree; 1 Somewhat agree; 2 Agree; 3 Strongly agree*]	Survey
**Country Level Covariates**		
Population	Total Population as per last available data in millions.	World Bank
GDP per capita	GDP per capita (current $US). GDP per capita is a measure of a country's economic output that accounts for its number of people.	Work Bank
Unemployment Rate	Unemployment rate, projections for 2020 based on the first trimester of 2020. The unemployment rate is the share of the labor force that is jobless, expressed as a percentage.	International Monetary Fund
Old age dependency ratio	Age dependency ratio, 65 and above. The demographic old-age dependency ratio is defined as the number of individuals aged 65	Work Bank

	and over per 100 people of working age defined as those aged between 20 and 64.	
Gini Index	The Gini index measures the extent to which the distribution of income (or, in some cases, consumption expenditure) among individuals or households within an economy deviates from a perfectly equal distribution.	Work Bank
Life Expectancy	Life expectancy at birth in years. Average number of years that a newborn is expected to live if current mortality rates continue to apply.	World Bank
Health expenditure	Current health expenditure (as % of GDP). General government health expenditure and private health expenditure in a given year, calculated as percentage of the GDP.	Work Bank
Private Health Expenditure	Domestic private health expenditure (% of current health expenditure). Share of current health expenditures funded from domestic private sources. Domestic private sources include funds from households, corporations and non-profit organizations. Such expenditures can be either prepaid to voluntary health insurance or paid directly to healthcare providers.	Work Bank
Out-of-pocket payments	Out-of-pocket payments (as % of current health expenditure). Out-of-pocket payments are defined as direct payments made by individuals to health care providers at the time of service use. This excludes any prepayment for health services, and it relies on user fees.	Work Bank
Hospital Beds	Hospital beds (per 1,000 people).	Work Bank
Cases per million	Confirmed COVID-19 cases per million people. Point estimate selected when data collection reached 50% of sample size completion each country. This corresponded to April 12th for Argentina, Brazil, Canada, France, Germany, Italy, Japan, South Africa, Spain, Turkey, UK and USA. This corresponded to April 20th for Australia, China, Greece, Indonesia, Netherlands, Philippines, Romania, Russia, Saudi Arabia, Serbia, South Korea and Ukraine.	Our world in data
Deaths per million	Confirmed COVID-19 deaths per million people. The point estimate selected for the numbers of deaths is equivalent to the description above about the number of cases.	Our world in data

Table S2. Sample Description

	ARGENTINA	AUSTRALIA	BRAZIL	CANADA	CHINA	FRANCE
Sample Size	1176	1030	1098	1053	1174	1089
Age	38%18-34;<br>33% 35-54<br>29% 55+	30%18-34<br>38% 35-54<br>32% 55+	42%18-34<br>36% 35-54<br>22% 55+	29%18-34<br>39% 35-54<br>32% 55+	49%18-34<br>51% 35-54<br>0% 55+	26%18-34<br>35% 35-54<br>39% 55+
Gender (% female)	53.2%	50.7%	53.2%	51.3%	50.6%	52.8%
Education	Up to higher education 70%; 30% University degree +	Up to higher education 60%; 40% University degree +	Up to higher education 70%; 30% University degree +	Up to higher education 59%; 41% University degree +	Up to higher education 51%; 49% University degree +	Up to higher education 73%; 27% University degree +
Employment Status	63% employed	55% employed	63% employed	56% employed	77% employed	52% employed
Financial Insecurity	Difficulty pay bills 46%	Difficulty pay bills 30%	Difficulty pay bills 41%	Difficulty pay bills 27%	Difficulty pay bills 22%	Difficulty pay bills 25%
Exposure COVID-19	.17 (.49)	.12 (.34)	.29 (.52)	.16 (.42)	.18 (.52)	.43 (.61)
Knowledge COVID-19	3.74 (.86)	3.47 (.82)	3.89 (.87)	3.62 (.85)	3.30 (.76)	3.82 (.85)
Knowledge economic loss	3.61 (1.07)	3.17 (.86)	3.65 (.94)	3.29 (.90)	2.59 (.78)	3.22 (.99)
Quality Messages	4.64 (1.39)	4.20 (1.32)	4.46 (1.43)	4.50 (1.27)	5.10 (.085)	3.97 (1.51)
Community Norms	1.94 (1.52)	1.48 (1.35)	1.02 (1.70)	1.60 (1.35)	1.67 (1.33)	0.86 (1.59)
Total Population	44.5	24.9	209.5	37.1	1392.7	66.9
GDP per capita	11684	57374	8921	46233	9771	41464
Unemployment Rate	10.9	7.6	14.7	7.5	4.3	10.4
Old Age Dependency Ratio	17	24	13	26	15	32
Gini Index	41.4	34.4	53.9	33.8	38.5	31.6
Life Expectancy	77	83	76	82	77	83
Health Expenditure	9.12	9.21	9.47	10.57	5.15	11.31
Private Health Expenditure	27.07	31.09	58.05	26.28	43.3	16.62
Out-of-pocket	15.02	18.15	27.46	14.22	36.05	9.38
Hospital Beds	5	3.8	2.2	2.7	4.2	6.5
COVID-19 Cases	44.30	257.92	91.30	584.51	58.12	1382.90
COVID-19 Deaths	1.84	2.69	4.89	15.29	3.22	200.38

Table S2. Sample Description (Cont.)

	**GERMANY**	**GREECE**	**INDONESIA**	**ITALY**	**JAPAN**	**NETHERLANDS**
Sample Size	1079	986	954	1115	1089	1041
Age	24%18-34<br>34% 35-54<br>42% 55+	24%18-34<br>38% 35-54<br>38% 55+	47%18-34<br>41% 35-54<br>12% 55+	23%18-34<br>37% 35-54<br>40% 55+	27%18-34<br>31% 35-54<br>42% 55+	26%18-34<br>37% 35-54<br>37% 55+
Gender (% female)	52.4%	48.2%	45.4%	52.3%	50.4%	50.7%
Education	Up to higher education 78%; 22% University degree +	Up to higher education 40%; 60% University degree +	Up to higher education 54%; 46% University degree +	Up to higher education 66%; 34% University degree +	Up to higher education 49%; 51% University degree +	Up to higher education 78%; 22% University degree +
Employment Status	54% employed	52% employed	68% employed	49% employed	56% employed	57% employed
Financial Insecurity	Difficulty pay bills 20%	Difficulty pay bills 43%	Difficulty pay bills 48%	Difficulty pay bills 25%	Difficulty pay bills 18%	Difficulty pay bills 16%
Exposure COVID-19	.21 (.44)	.16 (.42)	.44 (.63)	.37 (.57)	.12 (.33)	.45 (.70)
Knowledge COVID-19	3.76 (.76)	4.02 (.80)	3.44 (.84)	3.87 (.79)	2.96 (.86)	3.54 (.82)
Knowledge economic loss	3.37 (.87)	3.54 (.94)	3.33 (.88)	3.46 (.87)	2.74 (.87)	2.96 (.87)
Quality Messages	4.20 (1.34)	4.72 (1.29)	4.64 (1.36)	4.27 (1.36)	3.17 (1.19)	4.32 (1.22)
Community Norms	0.62 (1.62)	1.37 (1.31)	1.77 (1.29)	1.24 (1.38)	0.90 (1.33)	1.12 (1.40)
Total Population	82.9	10.7	267.7	60.4	126.5	17.2
GDP per capita	47603	20324	3894	34483	39290	53024
Unemployment Rate	3.9	22.3	7.5	12.7	3.0	6.5
Old Age Dependency Ratio	33	34	9	36	46	30
Gini Index	31.9	34.4	39	35.9	32.9	28.5
Life Expectancy	81	81	72	83	84	82
Health Expenditure	11.25	8.04	2.99	8.84	10.94	10.1
Private Health Expenditure	15.49	39.64	51.1	26.1	15.91	18.45
Out-of-pocket	12.67	34.75	34.61	23.49	12.85	11.09
Hospital Beds	8.3	4.3	1.2	3.4	13.4	4.7
COVID-19 Cases	1399.08	212.64	22.85	2444.93	44.18	1842.11
COVID-19 Deaths	30.20	10.23	1.99	312.05	0.71	209.01

Table S2. Sample Description (Cont.)

	PHILLIPINES	ROMANIA	RUSSIA	SAUDI ARABIA	SERBIA	SOUTH AFRICA
Sample Size	1029	1040	1040	980	1044	1151
Age	48%18-34<br>34% 35-54<br>17% 55+	29%18-34<br>36% 35-54<br>35% 55+	29%18-34<br>35% 35-54<br>36% 55+	54%18-34<br>42% 35-54<br>5% 55+	28%18-34<br>37% 35-54<br>35% 55+	46%18-34<br>32% 35-54<br>23% 55+
Gender (% female)	50.1%	51.6%	54.5%	40.8%	51.5%	51.6%
Education	Up to higher education 32%; 68% University degree +	Up to higher education 44%; 56% University degree +	Up to higher education 78%; 22% University degree +	Up to higher education 36%; 64% University degree +	Up to higher education 63%; 37% University degree +	Up to higher education 66%; 34% University degree +
Employment Status	53% employed	54% employed	55% employed	68% employed	54% employed	58% employed
Financial Insecurity	Difficulty pay bills 54%	Difficulty pay bills 29%	Difficulty pay bills 42%	Difficulty pay bills 41%	Difficulty pay bills 39%	Difficulty pay bills 50%
Exposure COVID-19	.30 (.59)	.17 (.40)	.14 (.38)	.32 (.62)	.32 (.55)	.13 (.35)
Knowledge COVID-19	3.61 (.85)	3.77 (.80)	3.89 (.84)	3.91 (1.07)	3.96 (.79)	3.72 (.88)
Knowledge economic loss	3.35 (.88)	3.44 (.90)	3.21 (.1.05)	3.43 (.1.15)	3.53 (.91)	3.44 (.95)
Quality Messages	4.45 (1.29)	4.54 (1.40)	4.35 (1.49)	4.84 (1.45)	4.33 (1.53)	4.63 (1.35)
Community Norms	1.83 (1.37)	1.14 (1.50)	0.56 (1.50)	1.60 (1.59)	1.41 (1.38)	1.67 (1.51)
Total Population	106.6	19.5	144.5	33.7	6.9	57.8
GDP per capita	3103	12301	11289	23339	7247	6374
Unemployment Rate	6.2	10.1	4.9	6.0	13.4	35.3
Old Age Dependency Ratio	8	28	22	5	28	8
Gini Index	44.4	36	37.5	45.9	36.2	63
Life Expectancy	71	75	73	75	76	64
Health Expenditure	4.45	5.16	5.34	5.23	8.11	8.11
Private Health Expenditure	65.5	21.41	42.91	35.91	43.13	44.39
Out-of-pocket	53.05	20.49	40.49	16.86	41.75	7.77
Hospital Beds	1.00	6.30	8.20	2.70	5.70	2.80
COVID-19 Cases	55.44	437.18	255.03	237.24	881.85	33.53
COVID-19 Deaths	3.63	21.68	2.16	2.64	16.17	0.38

Table S2. Sample Description (Cont.)

	**SOUTH KOREA**	**SPAIN**	**TURKEY**	**UK**	**UKRAINE**	**USA**
Sample Size	1021	1048	1068	1096	979	1055
Age	35%18-34<br>41% 35-54<br>24% 55+	22%18-34<br>39% 35-54<br>39% 55+	42%18-34<br>37% 35-54<br>22% 55+	29%18-34<br>35% 35-54<br>36% 55+	31%18-34<br>37% 35-54<br>32% 55+	30%18-34<br>37% 35-54<br>33% 55+
Gender (% female)	51.2%	51.1%	51.7%	52.1%	51.2%	52.9%
Education	Up to higher education 32%; 68% University degree +	Up to higher education 62%; 38% University degree +	Up to higher education 37%; 63% University degree +	Up to higher education 68%; 32% University degree +	Up to higher education 71%; 29% University degree +	Up to higher education 61%; 39% University degree +
Employment Status	65% employed	56% employed	58% employed	53% employed	56% employed	50% employed
Financial Insecurity	Difficulty pay bills 36%	Difficulty pay bills 32%	Difficulty pay bills 42%	Difficulty pay bills 22%	Difficulty pay bills 50%	Difficulty pay bills 36%
Exposure COVID-19	.23 (.47)	.61 (.73)	.52 (.68)	.34 (.58)	.13 (.34)	.28 (.52)
Knowledge COVID-19	3.76 (.85)	3.61 (.91)	4.05 (.86)	3.38 (.87)	3.81 (.84)	3.59 (.92)
Knowledge economic loss	3.47 (.83)	3.41 (1.08)	3.68 (1.00)	2.92 (.94)	3.14 (1.01)	3.35 (1.00)
Quality Messages	4.37 (1.24)	4.27 (1.40)	4.41 (1.40)	4.33 (1.38)	4.18 (1.47)	4.19 (1.45)
Community Norms	1.69 (1.33)	1.72 (1.56)	1.00 (1/67)	1.42 (1.48)	0.71 (1.53)	1.45 (1.50)
Total Population	51.6	46.8	82.3	66.5	44.6	326.7
GDP per capita	31363	30371	9370	42944	3095	62795
Unemployment Rate	4.5	20.8	17.2	10.1	4.8	10.4
Old Age Dependency Ratio	20	29	13	29	24	24
Gini Index	31.6	34.7	41.9	34.8	26.1	41.4
Life Expectancy	83	83	77	81	72	79
Health Expenditure	7.6	8.87	4.22	9.63	7	17.06
Private Health Expenditure	41.13	29.38	22.29	20.58	54.3	15.47
Out-of-pocket	33.67	23.57	17.38	15.96	52.32	10.99
Hospital Beds	11.50	3.00	2.70	2.80	8.80	2.90
COVID-19 Cases	207.97	3589.04	559.16	1052.45	123.97	1508.09
COVID-19 Deaths	4.56	338.18	11.92	156.96	3.24	56.47

Table S3. Summary statistics for risk perceptions [Means (SD), correlation, paired samples t-test]

	**Perceived Health Risk**	**Perceived Economic Risk**	***r***	**Paired samples t-test**		
				t	***df***	***p-value***
Argentina	3.41 (1.38)	4.68 (1.83)	.301	-22.42	1169	.000
Australia	3.29 (1.34)	4.29 (1.81)	.283	-16.06	1028	.000
Brazil	3.19 (1.48)	4.44 (1.81)	.323	-21.31	1092	.000
Canada	3.40 (1.27)	4.38 (1.78)	.302	-17.09	1050	.000
China	2.41 (1.27)	4.28 (1.85)	.345	-34.81	1173	.000
France	3.73 (1.41)	4.04 (1.70)	.329	-5.58	1082	.000
Germany	3.29 (1.31)	3.51 (1.76)	.270	-3.79	1077	.000
Greece	3.66 (1.36)	5.03 (1.68)	.209	-22.25	983	.000
Indonesia	3.04 (1.47)	4.71 (1.86)	.362	-26.92	940	.000
Italy	3.25 (1.23)	4.32 (1.66)	.249	-19.83	1112	.000
Japan	3.99 (1.14)	4.63 (1.54)	.419	-14.10	1085	.000
Netherlands	3.54 (1.31)	3.67 (1.56)	.323	-2.51	1038	.000
Philippines	2.36 (1.29)	4.14 (1.92)	.393	-30.78	1024	.000
Romania	2.94 (1.49)	4.20 (1.78)	.397	-22.25	1035	.000
Russia	2.87 (1.19)	4.36 (1.76)	.298	-26.61	1033	.000
Saudi Arabia	2.97 (1.53)	3.88 (1.87)	.329	-14.35	974	.000
Serbia	2.66 (1.22)	4.07 (1.75)	.276	-24.73	1035	.000
South Africa	3.09 (1.39)	5.04 (1.89)	.265	-32.67	1145	.000
South Korea	2.90 (1.49)	4.41 (1.77)	.347	-25.74	1018	.000
Spain	3.73 (1.44)	4.63 (1.68)	.265	-15.17	1046	.000
Turkey	3.57 (1.43)	4.72 (1.69)	.311	-20.24	1061	.000
UK	3.85 (1.40)	4.11 (1.79)	.306	-4.55	1094	.000
Ukraine	2.89 (1.25)	4.54 (1.68)	.334	-29.88	972	.000
USA	3.35 (1.56)	4.31 (1.90)	.411	-16.44	1051	.000

Figure S1. Standardized health perceived risk per country

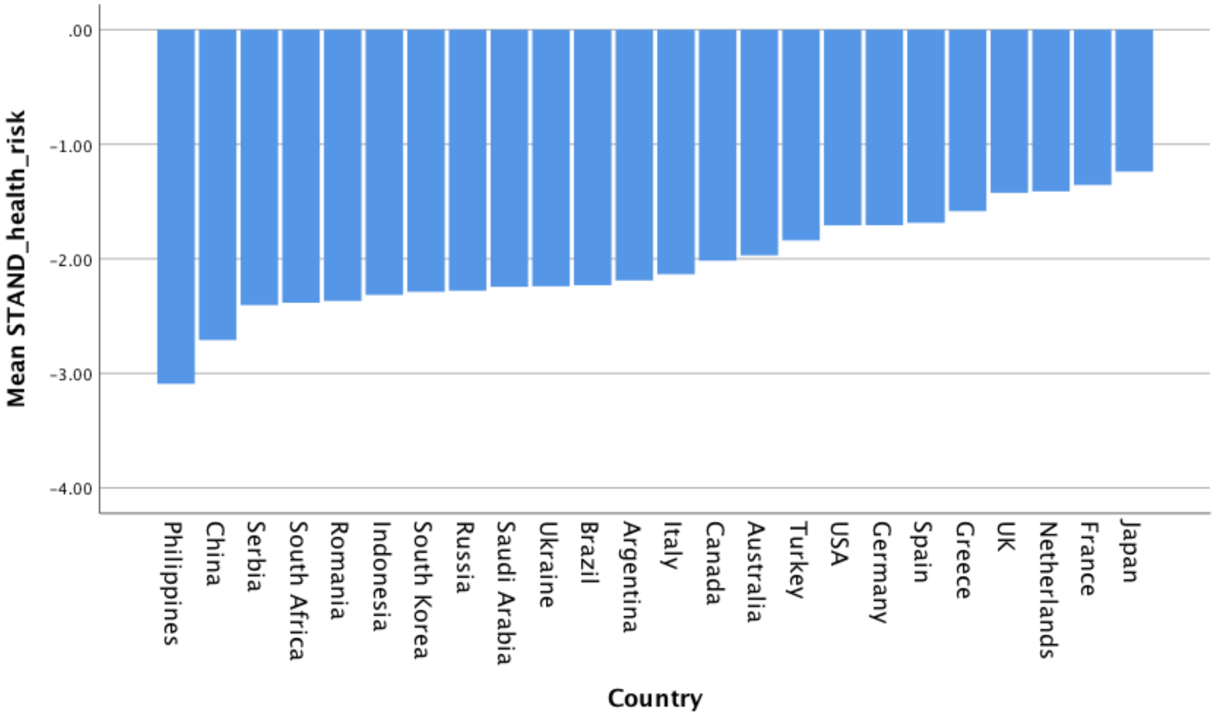

Figure S2. Standardized economic perceived risk per country

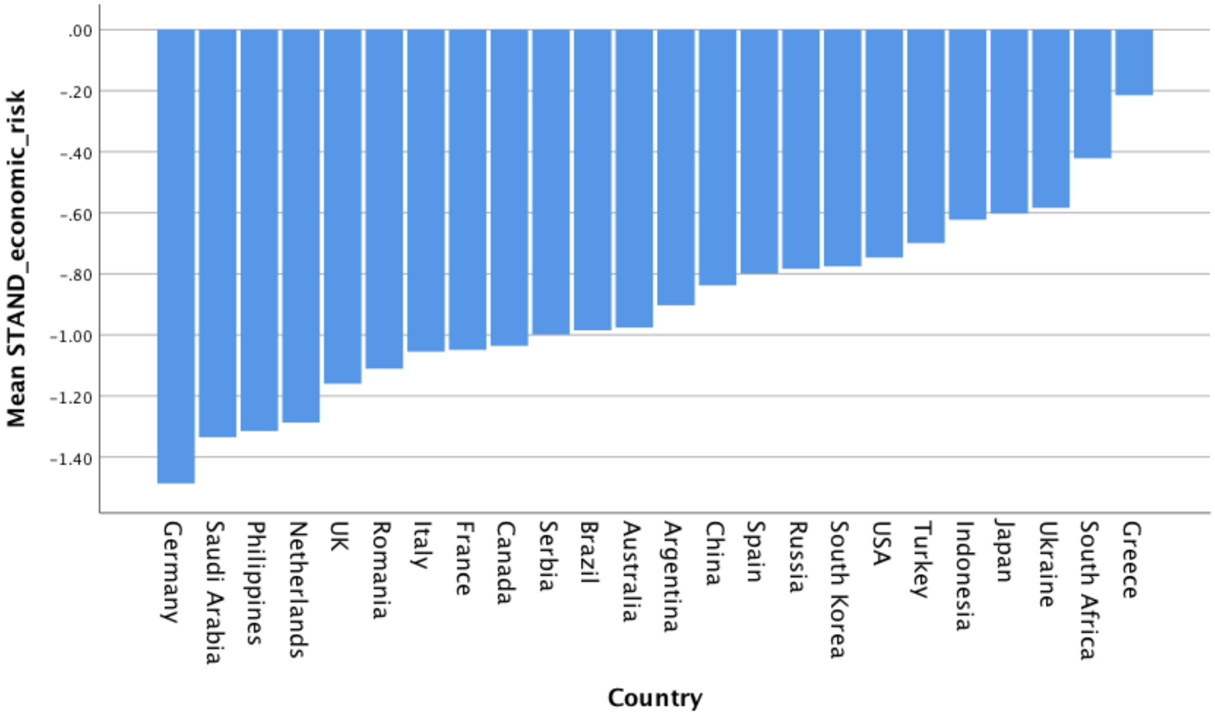

Figure S3. Perceived health and economic risk per sociodemographic groups (unadjusted means)

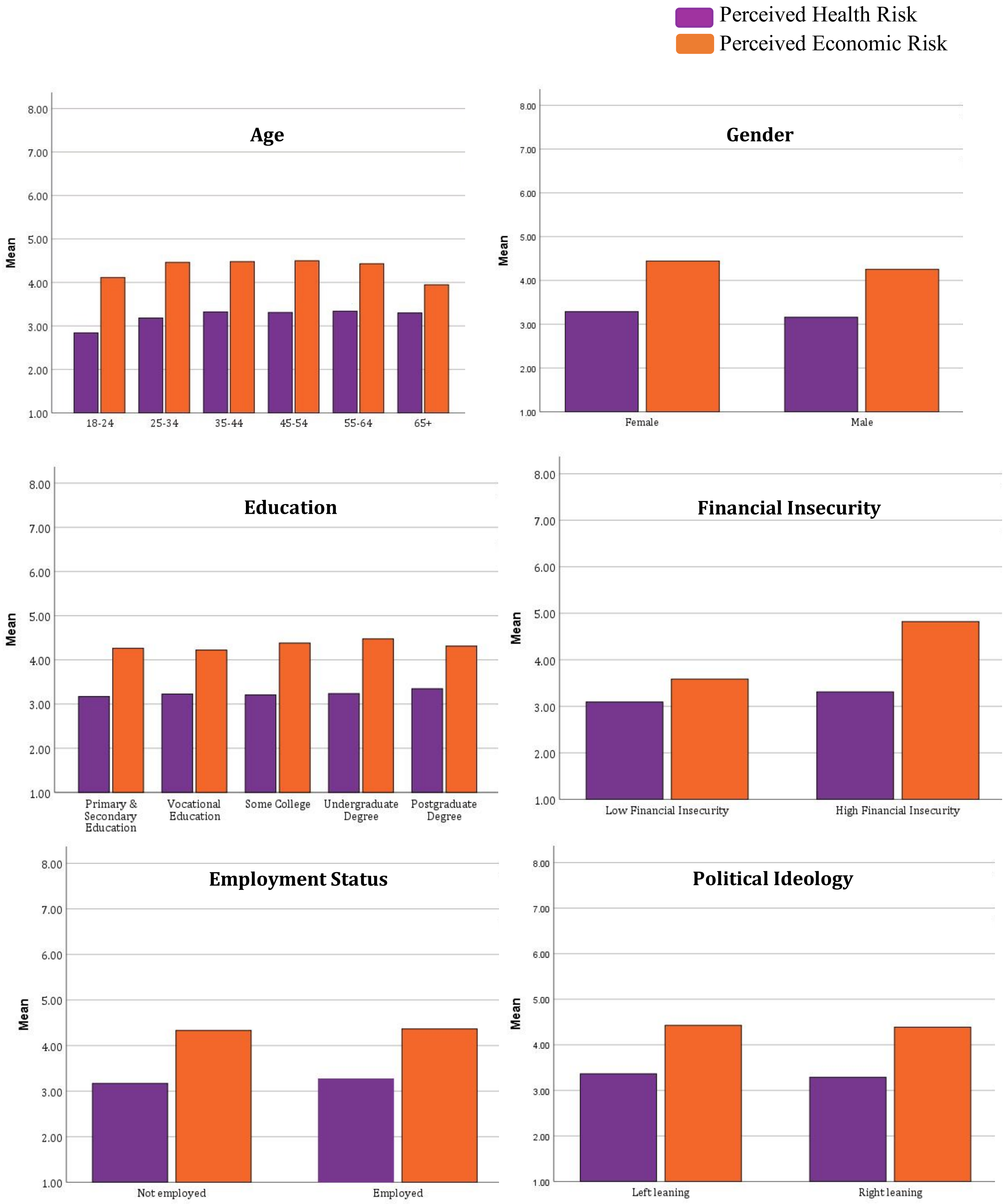

Per age groups, individuals aged 18-24 reported the lowest health risk perception (M=2.84 SD=1.50 95% CI 2.79-2.89), and lower than the group 25-34 (M=3.18 SD=1.52 95% CI 3.14-3.23; Tukey HSD *post-hoc* test p<.001). Individuals in older groups (35-65+) report a similar health risk perception (M=3.30 to M=3.34; all Tukey HSD *post-hoc* test p>.05), and higher than both younger groups. Regarding perceived economic risk, the oldest group 65+ has the lowest risk perception (M=2.95 SD=1.66 95% CI 3.89-4.00), and lower than the youngest 18-24 group (M=4.11 SD=1.92 95% CI 4.05-4.18; Tukey HSD *post-hoc* test p<.001). All ages groups representing the working force (25-64 years old) share a similar economic risk perception (M=4.43 to M=4.50; all Tukey HSD *post-hoc* test p>.05).

Gender comparisons show that women have a higher risk perception concerning both their health (M=3.29 SD=1.42 95% CI 3.27-3.32 vs. men health risk M=3.16 SD=1.43 95% CI 3.13-3.18 p<.001) and their economic situation (M=4.44 SD=1.79 95% CI 4.41-4.47 vs. men M=4.25 SD=1.81 95% CI 4.22-4.28, p<.001).

With respect to education attainment, the most educated group at the postgraduate level reports the highest health risk perception (M=3.35 SD=1.43 95% CI 3.21-3.25), compared to all other educational groups (Tukey HSD *post-hoc* tests p<.001). Among all these groups, the lowest economic risk perception is reported by the less educated individuals, both with primary and secondary education (M=4.26 SD=1.86 95% CI 4.21-4.32) and vocational education (M=4.22 SD=1.83 95% CI 4.16-4.28, p=.825).

Concerning employment status, employed (vs. unemployed) people perceive a higher risk to contract the virus (M=3.27 SD=1.46 95% CI 3.25-3.29; unemployed M=3.17 SD=1.38 95% CI 3.14-3.20, p<.001). Interestingly, there are no significant differences between employed and unemployed people about perceived economic risk: all share a similar concern (M=3.33 and M=3.37 *p*=.120).

More than individuals' employment status, it seems to be their financial situation that carries a greater weigh in risk perceptions. Individuals experiencing financial difficulties not only have a higher perceived health risk (M=3.31 SD=1.45 95% CI 3.29-3.33 vs. low financial difficulties M=3.09 SD=1.39 95% CI 3.07-3.12 p<.001) but a much higher risk economic perception (M=4.82 SD=1.75 95% CI 4.79-4.85 vs. low financial difficulties M=3.58 SD=1.62 95% CI 3.55-3.62 p<.001).

Lastly, participants that position themselves on the left (vs. right) side of the political spectrum have a higher perceived health risk (M=3.36 SD=1.38 95% CI 3.33-3.39; right side M=3.29 SD=1.42 95% CI 3.26-3.32, p=.001) but different political ideologies share the same level of perceived economic risk (M=4.43 and M=4.39 *p*=.138).

Table S4. Summary statistics for primary and secondary outcomes [Means (SD)]

	Frequent Hand Wash	Avoid Crowds	Social Isolation	Mandatory Vaccination	Mandatory Quarantine	Report Suspect Cases
Argentina	2.23 (1.39)	2.39 (1.24)	5.95 (1.88)	1.75 (1.75)	2.19 (1.37)	1.74 (1.57)
Australia	1.99 (1.26)	2.19 (1.12)	5.05 (2.39)	1.28 (1.69)	1.94 (1.41)	1.39 (1.52)
Brazil	2.33 (1.15)	2.42 (1.05)	4.73 (2.39)	1.95 (1.47)	2.15 (1.28)	1.44 (1.60)
Canada	2.21 (1.19)	2.36 (1.09)	5.61 (2.17)	1.40 (1.77)	2.10 (1.39)	1.44 (1.54)
China	2.49 (.83)	2.43 (.85)	3.16 (2.27)	1.08 (1.49)	2.13 (1.10)	2.10 (1.02)
France	2.08 (1.31)	2.26 (1.22)	5.36 (2.30)	0.97 (1.84)	1.54 (1.51)	0.32 (1.85)
Germany	1.91 (1.47)	2.15 (1.32)	5.33 (2.22)	1.16 (1.88)	1.51 (1.61)	0.55 (1.82)
Greece	2.24 (1.16)	2.33 (1.09)	4.90 (2.26)	0.31 (2.10)	1.96 (1.42)	0.96 (1.75)
Indonesia	2.38 (1.05)	2.34 (1.05)	3.39 (2.48)	1.83 (1.31)	2.16 (1.17)	2.01 (1.16)
Italy	2.09 (1.23)	2.42 (1.05)	5.51 (2.22)	1.51 (1.73)	2.11 (1.31)	1.43 (1.59)
Japan	1.89 (1.22)	1.99 (1.21)	5.27 (2.25)	1.19 (1.39)	1.26 (1.34)	1.06 (1.38)
Netherlands	1.74 (1.42)	1.98 (1.24)	4.87 (2.29)	1.08 (1.82)	1.56 (1.46)	0.64 (1.74)
Philippines	2.55 (0.95)	2.61 (0.86)	4.69 (2.45)	2.00 (1.41)	2.42 (1.08)	2.22 (1.14)
Romania	2.38 (1.08)	2.42 (0.99)	5.31 (2.14)	0.91 (2.05)	2.07 (1.38)	1.66 (1.55)
Russia	2.16 (1.19)	2.07 (1.21)	4.84 (2.42)	0.88 (1.82)	2.08 (1.26)	1.35 (1.59)
Saudi Arabia	2.05 (1.55)	2.22 (1.37)	4.36 (2.59)	1.67 (1.68)	1.94 (1.54)	1.92 (1.44)
Serbia	2.26 (1.11)	2.24 (1.21)	5.09 (2.10)	0.31 (2.09)	1.93 (1.34)	1.32 (1.70)
South Africa	2.34 (1.10)	2.50 (0.95)	5.26 (2.23)	1.00 (1.97)	2.11 (1.22)	1.76 (1.43)
South Korea	1.92 (1.26)	1.77 (1.26)	4.74 (2.03)	1.48 (1.35)	1.78 (1.30)	1.63 (1.30)
Spain	2.14 (1.41)	2.40 (1.22)	5.91 (1.97)	1.67 (1.62)	1.83 (1.42)	0.86 (1.77)
Turkey	2.18 (1.36)	2.28 (1.32)	4.84 (2.44)	1.41 (1.61)	1.85 (1.48)	1.84 (1.48)
UK	2.01 (1.36)	2.21 (1.31)	5.54 (2.23)	1.30 (1.74)	1.63 (1.54)	1.05 (1.68)
Ukraine	2.26 (1.12)	2.12 (1.15)	4.42 (2.38)	0.74 (1.92)	2.07 (1.21)	1.16 (1.61)
USA	2.05 (1.42)	2.17 (1.33)	4.80 (2.50)	0.84 (1.99)	1.54 (1.64)	0.76 (1.84)
Total M(SD)	2.17 (1.26)	2.27 (1.17)	4.97 (2.37)	1.25 (1.81)	1.92 (1.40)	1.37 (1.65)

Table S5. Summary statistics for primary and secondary outcomes (% agree and strongly agree)

	Preventive Health Behaviors			Support for Strict Health Policies		
	Frequent Hand Wash	Avoid Crowds	Social Isolation	Mandatory Vaccination	Mandatory Quarantine	Report Suspect Cases
Argentina	85%	87%	77%	73%	84%	71%
Australia	75%	80%	60%	54%	72%	55%
Brazil	87%	88%	51%	76%	81%	58%
Canada	81%	85%	72%	59%	77%	56%
China	91%	90%	17%	47%	77%	78%
France	74%	81%	65%	44%	56%	29%
Germany	74%	81%	62%	54%	62%	35%
Greece	84%	87%	53%	37%	75%	47%
Indonesia	88%	87%	27%	73%	82%	79%
Italy	79%	87%	69%	64%	80%	59%
Japan	71%	75%	61%	45%	46%	40%
Netherlands	71%	75%	53%	54%	64%	38%
Philippines	91%	93%	49%	76%	88%	83%
Romania	86%	88%	63%	49%	77%	64%
Russia	80%	76%	53%	44%	77%	55%
Saudi Arabia	79%	82%	45%	68%	75%	74%
Serbia	86%	85%	55%	37%	77%	58%
South Africa	85%	90%	61%	53%	80%	69%
South Korea	72%	67%	44%	55%	66%	61%
Spain	79%	85%	76%	66%	71%	44%
Turkey	82%	84%	54%	60%	75%	75%
UK	75%	80%	69%	55%	64%	46%
Ukraine	84%	80%	41%	43%	78%	50%
USA	78%	80%	54%	46%	62%	40%
Total Mean	81%	83%	55%	56%	73%	57%

Note: *Percentage of answers "agree" or "strongly agree" except social isolation, which presents answers for 6 or 7 days of social isolation in the past week.*

Figure S4. Association between health and economic risk, and each of the six outcomes (raw scores)

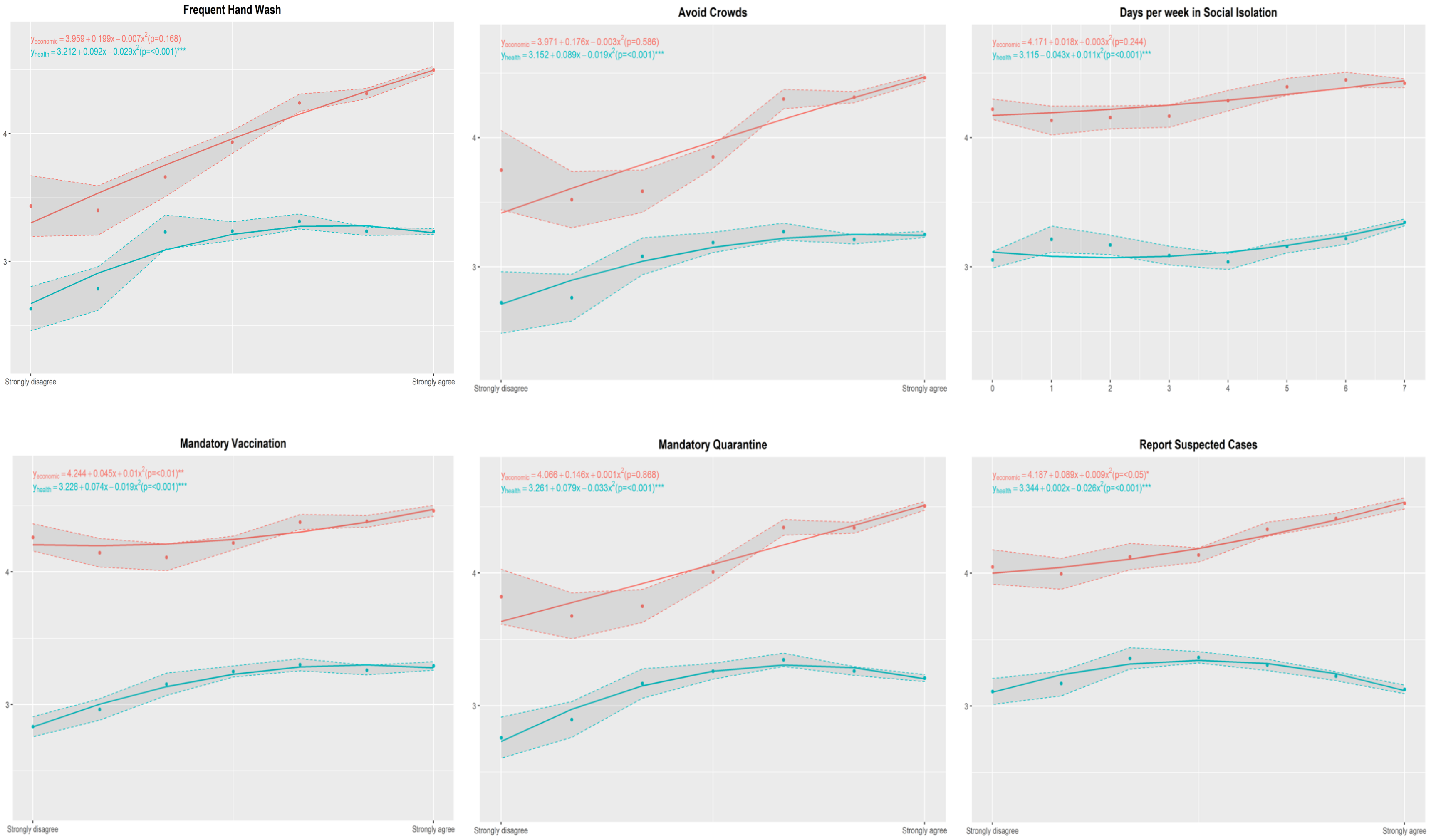

All these exploratory analyses plotting health and economic risk independently show both risks significantly predict all outcomes. However, multilevel regressions models (Table 1-2; Supplementary Table 6 and 7 below) show that health risk is no longer significant in several instances, when economic risk is taken into account. This suggests that part of the association between health risk and mitigation measures derives from fears of not being able to work/ income loss due to contracting the virus.

Table S6. Multilevel Regression Modeling: Preventive Health Behaviors

		Frequent Hand Wash			Avoid Crowds			Social Isolation		
		**0**	**1**	**2**	**0**	**1**	**2**	**0**	**1**	**2**
Main IVs	Intercept	.02 (.06)	.02 (.05)	.00 (.03)	-.01 (.06)	.00 (.06)	.01 (.03)	-.04 (.08)	-.08 (.23)	-.04 (.06)
	Control: Case-Fatality Rate	-.02 (.04)	-.02 (.04)	.00 (.02)	.01 (.04)	.00 (.04)	-.01 (.02)	.04 (.06)	.11 (.06)	.06 (.04)
	Health Risk (HR)		.01 (.02)	.03 (.02)		.00 (.02)	.02 (.01)		.01 (.01)	.01 (.01)
	Economic Risk (ER)		.11*** (.01)	.11*** (.01)		.09*** (.01)	.10*** (.01)		.05*** (.01)	.06*** (.01)
	HR X ER		.01 (.01)	.02** (.01)		.00 (.01)	.01 (.01)		-.02** (.01)	-.01 (.01)
	Health Risk^2(HR^2)		-.01 (.01)	-.01 (.00)		.00 (.01)	.01 (.00)		-.03*** (.01)	-.02** (.01)
	HR^2 X ER		.00 (.00)	-.01 (.00)		.01 (.00)	.00 (.00)		.00 (.00)	.01 (.00)
Individual-level covariates	Exposure Covid19			-.09*** (.01)			-.08*** (.01)			-.09*** (.01)
	Knowledge Virus			.18*** (.01)			.18*** (.01)			.04*** (.01)
	Knowledge Economy			-.01* (.01)			-.03*** (.01)			-.04** (.01)
	Quality Messages			.13*** (.01)			.13*** (.01)			.01 (.01)
	Community Norms			.21*** (.01)			.22*** (.01)			.05*** (.01)
	Age			.08*** (.01)			.07*** (.01)			.12*** (.01)
	Gender			-.07*** (.01)			-.07*** (.00)			-.08*** (.01)
	Education			.00 (.01)			.01** (.01)			.02*** (.01)
	Employment			-.01 (.01)			-.05*** (.01)			-.05*** (.01)
	Financial status			.00 (.01)			-.02*** (.01)			-.03*** (.05)
Country-level covariates	Total Population			.05* (.02)			.05 (.02)			-.18*** (.04)
	GDP per capita			-.18*** (.04)			-.09 (.05)			-.09 (.09)
	Unemployment Rate			-.05 (.03)			-.04 (.03)			-.03 (.05)
	Gini Index			-.03 (.04)			.01 (.05)			.12 (.08)
	Old Age Dependency Ratio			.14** (.04)			.15** (.05)			-.01 (.08)
	Life Expectancy			-.07*(.03)			-.08 (.04)			.06 (.07)
	Health Expenditure			.07* (.03)			.02 (.04)			.08 (.06)
	Private Health Expenditure			.08 (.06)			.04 (.07)			-.17 (.12)
	Out-of-pocket payment			-.08 (.06)			-.05 (.08)			.08 (.12)
	Hospital Beds			-.09** (.02)			-.14*** (.03)			-.02 (.05)

Table S7. Multilevel Regression Modeling: Support for Strict Containment Measures

		Mandatory Vaccination			Mandatory Quarantine			Report Suspected Cases		
		**0**	**1**	**2**	**0**	**1**	**2**	**0**	**1**	**2**
Main IVs	Intercept	-.09 (.08)	-.05 (.07)	-.04 (.06)	.00 (.07)	.01 (.07)	-.03 (.05)	-.02 (.10)	-.02 (.09)	-.06 (.07)
	Control: Case-Fatality Rate	.09 (.06)	.04 (.04)	.04 (.04)	.00 (.05)	-.01 (.05)	.03 (.04)	.02 (.07)	-.01 (.05)	.03 (.05)
	Health Risk (HR)		.05*** (.01)	.06*** (.01)		.01 (.01)	.03* (.01)		-.02 (.01)	.00 (.01)
	Economic Risk (ER)		.03* (.02)	.04** (.14)		.09*** (.01)	.10*** (.01)		.07*** (.01)	.05*** (.01)
	HR X ER		.01 (.01)	.01* (.01)		.00 (.01)	.00 (.01)		.01 (.01)	.01 (.01)
	Health Risk^2(HR^2)		.00 (.01)	.00 (.01)		.00 (.01)	.01 (.01)		.03*** (.01)	.02*** (.01)
	HR^2 X ER		.00 (.00)	.00 (.00)		.00 (.01)	.00 (.00)		.00 (.00)	.00 (.00)
Individual-level covariates	Exposure Covid19			-.01 (.01)			-.05*** (.01)			-.02* (.01)
	Knowledge Virus			.08*** (.01)			.16*** (.01)			.10*** (.01)
	Knowledge Economy			.02** (.01)			-.03*** (.01)			.01 (.01)
	Quality Messages			.13*** (.01)			.14*** (.01)			.14*** (.01)
	Community Norms			.15*** (.01)			.20*** (.01)			.16*** (.01)
	Age			.05*** (.01)			.06*** (.01)			-.01 (.01)
	Gender			.03*** (.01)			-.05*** (.00)			-.02*** (.01)
	Education			.00 (.01)			-.01 (.01)			-.04*** (.01)
	Employment			-.04*** (.01)			-.03*** (.01)			-.01 (.01)
	Financial status			-.02** (.01)			-.02** (.01)			.03*** (.05)
Country-level covariates	Total Population			-.03 (.03)			.00 (.03)			-.03 (.03)
	GDP per capita			-.07 (.07)			.00 (.06)			.08 (.09)
	Unemployment Rate			-.10* (.04)			-.03 (.04)			.01 (.05)
	Gini Index			.07 (.07)			.03 (.06)			.22* (.08)
	Old Age Dependency Ratio			.01 (.06)			.04 (.06)			-.03 (.08)
	Life Expectancy			.12*(.05)			-.05 (.05)			.06 (.06)
	Health Expenditure			.00 (.05)			-.02 (.05)			-.15 (.06)
	Private Health Expenditure			.14 (.10)			.02 (.09)			-.19 (.12)
	Out-of-pocket payment			-.06 (.10)			.06 (.09)			.28* (.12)
	Hospital Beds			-.05 (.04)			-.08* (.03)			-.03 (.05)

Table S8. Sensitivity Analysis: Multilevel Regression Modeling (Model 2) for low financial insecurity (n=8427)

	Hand Washing	Avoid Crowds	Social Isolation	Mandatory Vaccination	Mandatory Quarantine	Report Cases
Intercept	-.29* (.14)	-.20*** (.05)	-.27 (.07)	-.20* (.07)	-.16*** (.04)	-.04 (.11)
Control: Case-Fatality Rate	.04 (.04)	.01 (.02)	.02 (.03)	.04 (.03)	.01 (.01)	.03 (.03)
Health Risk (HR)	.04 (.03)	.04 (.03)	.04** (.01)	.06*** (.01)	.04* (.01)	-.02 (.02)
Economic Risk (ER)	.06* (.01)	.06** (.02)	.02 (.02)	.04 (.02)	.07*** (.01)	.05 (.03)
HR X ER	.01 (.25)	.00 (.03)	.02 (.03)	.00 (.02)	-.01 (.12)	-.01 (.05)
Health Risk2(HR2)	-.01 (.01)	-.01 (.01)	.01 (.01)	.00 (.04)	.00 (.01)	.02 (.01)
HR2 X ER	-.01 (.02)	.00 (.02)	.00 (.01)	-.01 (.01)	.00 (.11)	.00 (.01)
Adjusted ICC	.80	.11	.10	.18	.76	.27

Note: reporting unstandardized coefficients, standard errors in parentheses. $^{*}p<.05$, $^{**}p<.01$, $^{***}p<.001$. All predictors are presented in the Methods section and detailed in Table S1. The models controlled for COVID-19 case-fatality rate: total COVID-19 deaths per million/ total COVID-19 cases per million. Model 2 adjusted for individual and country level covariates as follows. Individual level covariates: (i) direct exposure to someone in their personal network (self, family, friends) infected with COVID-19; (ii) perceived knowledge about the COVID-19, (iii) perceived knowledge about the economic consequences of the COVID-19; (iv) the perceived quality of the public messages received, (v) community norms about mitigation measures, and (vi) sociodemographic variables (age, gender, education, employment and financial status). Country-level covariates included (i) total population of the country (in millions), (ii) gross domestic product (GDP) per capita (in current $US), (iii) unemployment rate estimates for 2020 (as % of the labor force), (iv) old age dependency ratio (%), (v) Gini Index, (vi) general health expenditure (as %GDP), (viii) private health expenditure (as % health expenditure), (viii) out-of-pocket health payments (as % health expenditure), (ix) number of hospital beds (per 1000 people).

Table S9. Fixed-effects Model

	Hand Washing	Avoid Crowds	Social Isolation	Mandatory Vaccination	Mandatory Quarantine	Report Cases
Health Risk (HR)	.03***(.01)	.02***(.01)	-.01(.00)	.06***(.01)	.03***(.01)	.00(.01)
Economic Risk (ER)	.1***(.01)	.02***(.01)	-.06***(.01)	.04***(.01)	.09***(.01)	.05***(.01)
HR X ER	.02**(.01)	.01(.01)	.01(.01)	.01*(.00)	.00(.00)	.01(.01)
Health Risk^2(HR^2)	-.01 **.00)	-.01*(.00)	.02**(.01)	.00(.00)	.00(.00)	.02***(.00)
HR^2 X ER	-.00(.00)	.00(.00)	.00(.00)	.00(.00)	.00(.00)	.00(.00)

. $^{*}p<.05$, $^{**}p<.01$, $^{***}p<.001$

Table S10. Lasso Model

	Hand Washing	Avoid Crowds	Social Isolation	Mandatory Vaccination	Mandatory Quarantine	Report Cases
Intercept	-.19*** (.02)	-.16***(.01)	.25*** (.03)	-.11 * (0.05)	-.14 *** (.03)	-.01 (.04)
Health Risk (HR)	.02 (.02)	.02 * (.01)	-.01(.01)	.06 ***(.01)	.03 * (.01)	-.00 (.01)
Economic Risk (ER)	.11***(0.01)	.01*** (.01)	-.07***(.01)	.04**(.01)	.10*** (.01)	.05 ***(.01)
HR X ER	.01* (.00)	.01(.01)	.01 (.01)	.01*(.01)	.00 (.00)	.01(.01)
Health Risk^2(HR^2)	-.01 (.01)	-.01 (.01)	.02**(.01)	.00 (.01)	.00 (.01)	.02***(.01)
HR^2 X ER	-.01(.00)	.00 (.00)	.01(.01)	.00 (.00)	-.01 (.00)	-.00(.00)

. $^{*}p$<.05, ** p<.01, $^{***}p$<.001
